# Redox Homeostasis and Metabolism in Cancer: A Complex Mechanism and Potential Targeted Therapeutics

**DOI:** 10.3390/ijms21093100

**Published:** 2020-04-28

**Authors:** Alia Ghoneum, Ammar Yasser Abdulfattah, Bailey Olivia Warren, Junjun Shu, Neveen Said

**Affiliations:** 1 Departments of Cancer Biology, Wake Forest University School of Medicine, Winston Salem, NC 27157, USA; 2 Departments of Urology, Wake Forest University School of Medicine, Winston Salem, NC 27157, USA; 3 The Third Affiliated Hospital of Nanchang University, Nanchang 330006, China; 4 Comprehensive Cancer Center, Winston Salem, NC 27157, USA

**Keywords:** redox systems, ROS, oxidative stress, metabolism, HIF-1α, PGC-1α, Nrf2, metabolic targeting

## Abstract

Reactive Oxygen Species or “ROS” encompass several molecules derived from oxygen that can oxidize other molecules and subsequently transition rapidly between species. The key roles of ROS in biological processes are cell signaling, biosynthetic processes, and host defense. In cancer cells, increased ROS production and oxidative stress are instigated by carcinogens, oncogenic mutations, and importantly, metabolic reprograming of the rapidly proliferating cancer cells. Increased ROS production activates myriad downstream survival pathways that further cancer progression and metastasis. In this review, we highlight the relation between ROS, the metabolic programing of cancer, and stromal and immune cells with emphasis on and the transcription machinery involved in redox homeostasis, metabolic programing and malignant phenotype. We also shed light on the therapeutic targeting of metabolic pathways generating ROS as we investigate: Orlistat, Biguandes, AICAR, 2 Deoxyglucose, CPI-613, and Etomoxir.

## 1. Reactive Oxygen Species

Reactive oxygen species “ROS” encompass several molecules derived from oxygen which can oxidize other molecules and subsequently transition rapidly between species [1,2]. ROS are highly reactive, due to the presence of unstable bonds or unpaired valence electrons [2]. Cellular ROS exist as free radicals, (hydroxyl, OH^−^), neutral molecules (hydrogen peroxide, H_2_O_2_), or ions (superoxide anion, O_2_^−^) [2,3,4,5]. The one-electron reduction of molecular oxygen forms the short-lived superoxide O_2_^−^ as it dismutates, catalyzed by superoxide dismutase (SOD), forming more stable hydrogen peroxide (H_2_O_2_) that can diffuse across membranes [2]. H_2_O_2_ is the most abundant form of ROS found in eukaryotes [3]. Additionally, reactive nitrogen species (RNS) exist within the cell as peroxynitrite (ONOO^−^), nitric oxide (NO), and nitrogen dioxide (NO_2_) [3,6]. RNS are clearly linked to ROS, as evidenced by their crosstalk and the intersection in their generation, function, and turnover [7,8]. This is particularly evident in the reaction of O_2_^−^ and NO generating ONOO^−^ [7]. Moreover, an analogous relationship is observed among the thiol oxidation products involved in regulation and cell signaling with nitrosothiols formed from NO [7,8,9]. 

## 2. Sources of ROS

Intracellular ROS generation can be instigated by both exogenous and endogenous stimuli [10]. Exogenous stimuli include environmental stressors such as UV and ionizing radiations (gamma-ray/x-ray), pollutants, chemicals, heavy metals, as well as xenobiotics (e.g., drugs) [4,10]. Endogenous ROS production is driven by both nonenzymatic and enzymatic reactions [11]. Enzymatic reactions involved in the cytochrome P450 system, prostaglandin synthesis, respiratory chain, and phagocytosis all generate ROS as a metabolic byproduct [2,4], whereas nonenzymatic sources include redox-active metals such as copper (Cu) and iron (Fe) [10]. In addition, hydroxyl radicals are generated as a byproduct of the Fenton/Haber-Weiss reaction in which H_2_O_2_ oxidizes Fe(II) [1,3,10]. 

**Figure 1:**
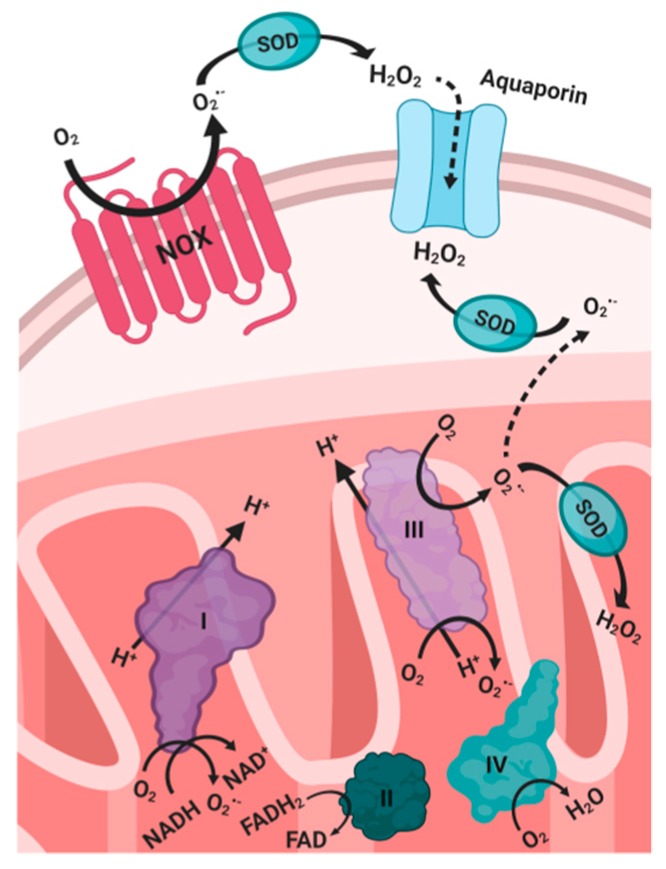
Primary generators of endogenous ROS. SOD: superoxide dismutase. NOX: nitric oxide oxidase. NAD: nicotinamide adenine dinucleotide. FAD: flavin adenine dinucleotide.

The primary generators of endogenous ROS are NADPH oxidase complexes (NOX) (Figure 1) and the mitochondrial electron transport chain (ETC) [12,13] through the production of superoxide in the mitochondrial ETC by electron leakage from Complex I (NADPH dehydrogenase), and in Complex III (ubiquinone-cytochrome c reductase) [3,6]. Electron and proton leaks occur due to inefficient coupling between phosphorylation and respiration [6]. In the ETC, electrons are transferred through the reduction of the electron carriers, flavin adenine dinucleotide (FADH2) and nicotinamide adenine dinucleotide (NADH) [6]. Subsequently, in Complex IV, cytochrome c oxidase (COX) catalyzes the tetravalent reduction of molecular oxygen (O_2_) into water as it acts as the final electron acceptor during oxidative phosphorylation [6]. 

NOX is a family of transmembrane enzymes that exists in several isoforms and is ubiquitous in various cell types [6]. ROS is generated during the activation of NOX in response to cytokines, growth factors, or G protein-coupled receptor (GPCR) agonists [6]. NOX produces ROS as it catalyzes the electron reduction of oxygen from the electron carrier NADPH, and its subsequent transfer through FAD and heme cofactors [3]. Superoxide generated during this process rapidly dismutates to H_2_O_2_, which can then translocate into the cell through specific aquaporin channels in the plasma membrane [6]. 

## 3. Physiological Roles of ROS

**Figure 2:**
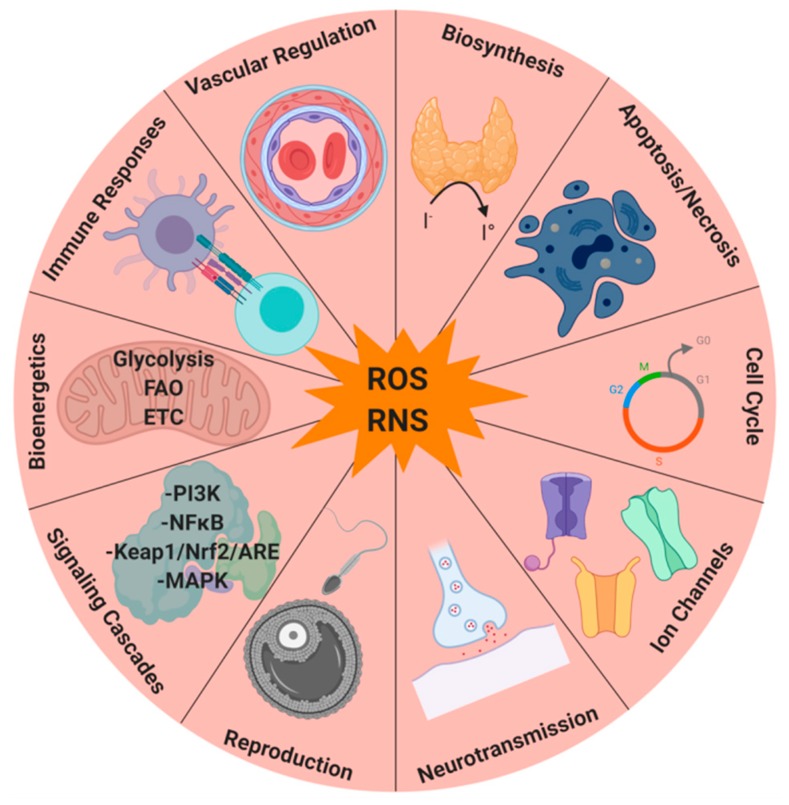
Physiological roles of ROS and RNS.

Many normal physiological processes and cellular functions rely on ROS and redox regulation [2]. Amino acids, especially cysteine, present in several proteins including phosphatases, kinases, receptors, transcription factors, and ion channels, can be redox-regulated and modified by ROS [2,7,14] (Figure 2). The duration, localization, and quantity of ROS generation ensure biological specificity [2]. Accordingly, many biological processes, such as cell differentiation, adaptive immunity, and oxygen sensing rely on ROS formed specifically from Complex III of the mitochondrial ETC [1]. Furthermore, ROS produced in the mitochondrial ETC Complex I has been associated with pathological conditions that arise with continually elevated cellular levels of ROS, such as inflammatory and immune system dysfunctions, allergies, neurodegenerative and cardiovascular disease, diabetes, aging, and cancer [6]. ROS can also act as signaling (Figure 2) molecules at low to moderate levels, and can intervene accordingly in the cell cycle (Figure 2) via cellular proliferation, growth, differentiation, apoptosis, migration, and cytoskeletal regulation [2,11]. Biosynthetic pathways (Figure 2), such as the crosslinking of extracellular matrix proteins and the iodination of thyroid hormone also rely on ROS [2]. Moreover, reproductive systems depend on ROS to facilitate fertilization and normal maturation [15,16,17] (Figure 2). In addition, ROS are important during host defenses, directly and indirectly contributing to the destruction of microorganisms [18,19,20,21,22] (Figure 2). Thus, the key roles of ROS in biological processes are cell signaling, biosynthetic processes, and host defense. 

## 4. Oxidative Stress

Under physiological conditions, there is equilibrium between intracellular exogenous, endogenous ROS and antioxidants as they regulate the generation and elimination of ROS [5,10]. The cellular redox balance is regulated by the relative concentrations of oxidized and reduced forms of enzymes, proteins, RNS, ROS, and thiol-containing molecules [5]. Accordingly, oxidative stress ensues in a biological system of cells and tissue, when there is an imbalance between the generation of ROS and the proficient removal of these elements [4,11]. Sustained elevation of ROS, particularly free radicals, causes damage to DNA, lipids, and proteins [2]. DNA oxidation leads to changes in gene expression, as well as DNA mutations [2]. Since the mitochondria lack DNA repair enzymes, mitochondrial DNA (mtDNA) is more sensitive to oxidative stress-induced mutations [1,2]. At the cellular level, superoxide is maintained at a steady-state concentration in the picomolar range, whereas H_2_O_2_ is maintained in the range of 1–10 nM [3]. However, minute and regulated deviations from these steady-state concentrations can trigger biological responses in the form of ROS signaling cascades [3], triggered as ROS are released into the cytosol from the mitochondria resulting in excess oxidative stress. In turn, ROS production increases transiently as the mitochondrial membrane potential is reduced, and subsequently elicits additional ROS release by neighboring mitochondria that may then induce cell death [3]. 

Many cytoprotective enzymes are activated and regulated at the transcriptional level by oxidative stress [6]. Antioxidant response elements (ARE) facilitate this transcriptional response. These ARE were originally discovered in the promoters of genes encoding NADPH quinone oxidoreductase-1 (NQO1) and glutathione S-transferase A2 (GSTA2), the two major detoxification enzymes [6]. In several cell types, the synthesis of antioxidant enzymes including glutathione peroxidase (GPx), thioredoxin reductase (TrxR), peroxiredoxin 1 (Prdx-1), heme-oxygenase-1 (HO-1), glutathione reductase (GR), and glutaredoxin (Grx) were found to be ARE-dependent [6]. Elevated ROS also activates the Nrf2/Keap1 (nuclear factor erythroid 2-related factor 2/ Kelch-like ECH-associated protein 1) pathway, which regulates an additional intracellular antioxidant defense where oxidative stress causes Nrf2 to translocate into the nucleus to bind with ARE located within the regulatory gene regions and consequently, regulate the expression of downstream target genes [6].

## 5. Oxidative Stress and Cancer

An observed hallmark of many tumors and cancer cell lines is the elevated rate of ROS scavenging to counterbalance the slightly higher levels of ROS present under normal physiological conditions. This phenomenon is referred to as “mild oxidative” stress, which is associated with the activation of oncogenic pathways [1,11]. Accordingly, ROS production by cancer cells results from modifications in numerous signaling pathways that affect cellular metabolism [23]. Oxidative stress can promote cancer progression via genome instability and chromosomal abnormalities with amplified oncogene activation, altered cancer cell metabolism, and loss of tumor suppressor genes [4,23,24]. DNA damage results in hydrolyzed DNA bases, forming adducts that impair the normal growth of cells through the induction of gene mutations and the alteration of normal physiological transcription [4,25,26]. Furthermore, a multitude of DNA mutations may result from oxidative stress in the form of DNA-protein crosslinks, the rearrangement of DNA sequences, base-free sites, gene duplications, the miscoding of DNA base and sugar subunits, and the activation of oncogenes, as well as strand breaks [2,4]. Moreover, excessive cellular ROS may accumulate in cancer cells due to the high metabolic rate in the endoplasmic reticulum, cell membrane, and in the mitochondria. Due to the fact that rapidly proliferating cancer cells require high levels of ATP to meet their elevated metabolic demands, continuous mitochondrial respiration is necessary [6,11]. Consequently, mitochondrial ETC and coupling efficiency are impaired leading to increased leakage of electrons [6] (Figure 1). Thus, cancer cell ROS-derived mutations promote further ROS production fostering cancer progression [6].

**Figure 3:**
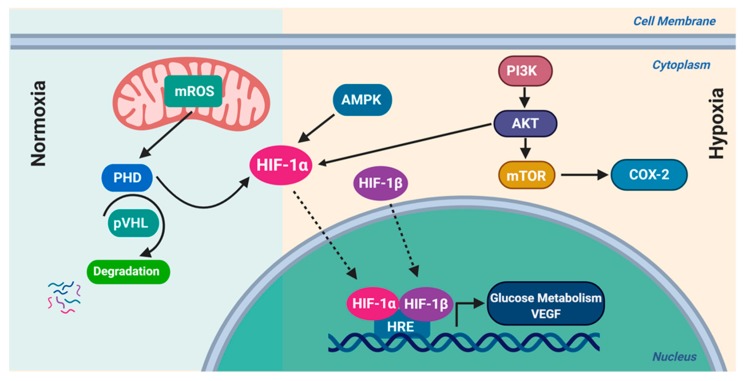
Activation of HIF-1α in normoxic and hypoxic conditions.

A hypoxic tumor microenvironment triggers the activation of various genes that regulate cell survival, proliferation, and growth [6], and consequently escalates the generation of ROS in tumors by HIF-1*α* (hypoxia-inducible factor 1 alpha) and its target genes [6]. Several cytokines and growth factors are generated during hypoxic exposure. The activation of these pathways upregulates NOX, increases ROS production, and hence activates downstream survival pathways [13,23,27]. High ROS levels promote tumorigenesis through the activation of myriad pathways such as the phosphatidylinositol-3 kinase (PI3K)/ protein kinase B (AKT)/ nuclear factor Kappa-light-chain-enhancer of activated B cells (NFκB) pathway (Figure 3). Furthermore, it has been reported that ROS contributes to cancer progression and survival by phosphorylating JUN N-terminal kinase (JNK), promoting expression of cyclin D1 and activating mitogen-activated Protein Kinase (MAPK) [24,27]. Moreover, an abundance of ROS levels affects cellular proliferation through the phosphorylation and activation of both extracellular-regulated kinase 1/2 (ERK1/2) and ligand-independent receptor tyrosine kinase (RTK), angiogenesis through the release of angiopoietin, vascular endothelial growth factor (VEGF), tissue invasion, and metastasis through the secretion of metalloproteinase (MMP) into the extracellular matrix. Additionally, such levels influence Rho-Rac interaction and the overexpression of Met oncogene [13,27]. ROS has been linked to several significant tumor metastasis processes including survival upon matrix detachment, loss of cell-to-cell adhesion, and migration and invasion through the cell basement membrane [28]. Several tumor suppressors are inactivated by ROS as they lead to the oxidation of cysteine residues at their catalytic sites; phosphatase and tensin homolog (PTEN) and protein tyrosine phosphatases (PTPs) are examples of tumor suppressors inactivated by ROS [24]. 

## 6. Metabolic Pathways and Redox Homeostasis

### 6.1. Glycolysis 

The most common glycolytic pathway was discovered in the 20^th^ century, where glucose is transported from the extracellular space to the cytosol by glucose transporters and converted to glucose-6-phosphate by hexokinases. Subsequently, a series of enzyme-catalyzed reactions occur, yielding two moles each of pyruvate, adenosine tri-phosphate (ATP), and NADH, per mole of glucose (summarized in [29]). In addition, Otto Warburg [30,31,32] reported that even in aerobic conditions cancer cells have a tendency to undergo glycolytic metabolism instead of the more efficient and preferred method, i.e., oxidative phosphorylation, a phenomenon that has since come to be known as the “Warburg effect” [30,31,32]. One invaluable determinant of cellular redox potential is the continuous supply of mitochondrial NADH that is necessary for electron transport [33]. Glucose metabolism is an essential determinant of redox homeostasis in tumors, as glycolytic intermediates are shuttled into the metabolic pathways that either directly or indirectly generate reducing equivalents, mainly pentose phosphate pathway (PPP)-derived NADPH or glutaminolysis-derived reduced glutathione (GSH) [34]. When glycolytic rates vary, several cellular mechanisms are in place to sustain redox homeostasis. One such mechanism is the malate-aspartate the shuttle of tricarboxylic acid (TCA) cycle, which allows electrons produced during glycolysis to pass the inner mitochondrial membrane; hence, it is aptly able to restore NADH imbalance. However, when the rate of glycolysis overwhelms the limits of the malate-aspartate shuttle, the conversion of pyruvate into lactate occurs via lactate dehydrogenase (LDH) with the production of NAD+ [35]. While the metabolic adaptations of cancer cells are highly complex, several promising attempts have been made to exploit glucose metabolism to target and ultimately inhibit cancer progression [36]. 

### 6.2. Fatty Acid Oxidation 

Fatty acid oxidation (FAO) is a series of measured oxidations that take place in the mitochondria which allows for long- and short-chain fatty acids to be truncated, leading to the generation of NADH, FADH2 and acetyl-CoA [37]. All three of these products are consequently used by a cell in bio-energetic pathways to produce ATP. A significant fraction of acetyl-CoA enters into the TCA cycle and generates citrate [29]. A portion of this citrate is then exported into the cytosol where ATP-citrate lyase (ACLY) breaks it down to oxaloacetate and acetyl-CoA [29]. NADPH can then be yielded by the oxidative decarboxylation of oxaloacetate to pyruvate by malic enzyme (ME) [29,37]. Alternatively, malate can be produced by the swift reduction of oxaloacetate, which is then reoxidized after being transported back to the mitochondria [29,37]. The generation of NADPH by FAO prevents cancer cell death during the loss of matrix adhesion and metabolic stress conditions through the modulation of the liver kinase B1 (LKB1)/AMPK axis [38]. Importantly, the key FAO regulators, such as the carnitine palmitoyltransferase-1 (CPT1), are overexpressed in solid and hematologic malignancies [39,40], and pharmacological inhibition of FAO impairs NADPH production, promotes oxidative stress-induced cell death and strengthens the proapoptotic effect of cytotoxic agents [41,42,43,44,45,46,47,48].

### 6.3. Pentose Phosphate Pathway 

The pentose phosphate pathway (PPP) is a key glucose catabolic pathway whereby cancer cells generate marked levels of ribose-5 phosphate, a precursor of nucleotide synthesis. Ribose-5 phosphate is also a critical substrate for anabolic processes to detoxify harmful ROS [49,50]. The overexpression of glucose-6-phosphate dehydrogenase (G6PD), the rate-limiting enzyme of the PPP, enhances the PPP-dependent production of NADPH [51]. The regulation of G6PD directly depends on the availability of glucose, as glucose funneling into the oxidative branch of the PPP directly controls the redox homeostasis [52]. TP53-induced glycolysis and apoptosis regulator (TIGAR) is a homologue of fructose-2,6-bisphosphatase [10] whose expression inhibits glycolysis, induces a shift toward the PPP through inhibition of phospho-fructokinase activity and consequently reduces the levels of fructose-2,6-bisphosphate [10]. This shift toward the PPP leads to the production of NADPH and reduced glutathione (GSH), the major ROS scavengers [10,53]. The downregulation of TIGAR expression by the synergistic effect of GO-203, a peptide inhibitor of oncoprotein mucin 1 C-terminal subunit (MUC-1), and bortezomib, a proteasome inhibitor, results in a decrease of GSH generation, inducing oxidative stress with ROS-mediated cell death in multiple myeloma cells [53]. 

### 6.4. Glutamine Metabolism 

Glutaminolysis is yet another pathway mediating the redox balance in cancer cells. As a nonessential amino acid, glutamine is a glutathione production precursor, an energy generation intermediate, and a nitrogen and carbon supply for nucleotide biosynthetic procedures [54]. An increase in glutamine catabolism commonly indicates a reprogramming of tumor metabolism that supports redox homeostasis, signal transduction, and cell proliferation [54]. Glutamine deprivation can decrease GSH levels in neuroblastoma cells, modifying the redox balance, impairing cell proliferation, and increasing their chemosensitivity to alkylating agents [55]. Glutamine can be converted into glutamate by glutaminase enzymes (GLS1/2); these enzymes contribute directly to glutathione synthesis and promote cysteine uptake [54]. GLS1 inhibition in P493 B-cell lymphoma (BCL) cells by bis-2-(5-phenylacetamido-1,3,4-thiadiazol-2-yl)ethyl sulfide (BPTES) has been shown to weaken cell proliferation, induce DNA fragmentation, and lead to apoptotic cell death. Accordantly, in mice with Myc-induced hepatocellular carcinoma, genetic silencing of GLS1 significantly impaired tumor growth and prolonged survival [56]. GLS1 inhibition by BPTES has also been found to selectively suppress the growth of cancer cells IDH1 and IDH2 mutations [57,58]. The inhibition of glutaminolysis has been linked with intracellular GSH content diminution and the ensuing production of ROS, particularly in carcinoma cells with glutamine addiction [59,60]. Finally, for cancer cells and mouse embryonic fibroblasts, synthetic lethality has been shown to be induced by GSL1 inhibition in combination with the inhibition of heat-shock protein 90, activating modifications to the mammalian target of the rapamycin complex I (mTORC1) pathway via increased ER stress and a depletion of GSH that disturbs redox balance [61]. 

### 6.5. The Serine–Glycine One-Carbon Metabolism (SGOC)

Cancer cells have long been linked with serine–glycine one-carbon metabolism (SGOC) due to the important role of SGOC in regulating protein synthesis, nucleic acids, and lipids in proliferating cells. SGOC is a complex biochemical reaction network that integrates input from glucose derivatives, mainly serine and glycine, as well as amino acids and generates carbon unit outputs (tetrahydrofolate (THF) and its derivate) [62]. Recently this pathway has also been shown to be crucial in redox balance; serine is primarily used in mammalian cells mitochondria for NADPH generation [62,63]. Serine catabolism, along with glycine, is responsible for mitochondrial generation of NADPH [62,63]. The expression of methylenetetrahydrofolate dehydrogenase 2 (MTHFD2), phosphoserine aminotransferase 1, and 3-phosphoglycerate dehydrogenase, pivotal one-carbon metabolism enzymes, [62,63] is regulated with by the antioxidant transcription factor Nrf2, supporting nucleotide and glutathione synthesis [64]. Further studies revealed that the use of antifolates such as methotrexate and pemetrexed represents a cornerstone of antineoplastic therapy against solid and hematologic malignancies [64,65]. Finally, pathways of one-carbon metabolism in chemo-resistant tumors, can be successfully targeted with agents that interfere with nucleotide synthesis such as 5-FU or gemcitabine [62]. 

### 6.6. Oxidative Phosphorylation 

Oxidative phosphorylation (OxPhos) is integral to the maintenance of redox homeostasis as it serves as a major supplier of ATP in cancer cells by the phosphorylation of ADP by electron transport in the mitochondria during aerobic respiration [66,67]. Mitochondria not only serve as the major energy source of the cell, they also produce superoxide anions by the hosted metabolic enzymes and multiple redox-active complexes. Hence, mitochondria are a significant endogenous ROS generation source [66,67]. In the ETC, the transfer of electrons from reduced metabolic intermediates, NADH and FADH2, to molecular oxygen occurs in a process that depends on oxygen availability and mitochondrial membrane potential status; univalent oxygen reduction into superoxide is supported by semiquinone radical generation at complexes I, II, and III [12,68,69,70]. Additional mechanisms for producing superoxide in mitochondria include the electron transfer flavoprotein-ubiquinone oxidoreductase mitochondrial system in the inner membrane, mitochondrial glycerol-3 phosphate dehydrogenase, pyruvate dehydrogenase in the mitochondrial matrix, and 2-oxoglutarate dehydrogenase [12,68,69,70].

## 7. Effects of Tumor Microenvironment (TME) Metabolism on Immune Cells and Immunotherapy

A discussion of the effect of tumor cell metabolism on surrounding immune cells in the TME, as well as the consequences this effect may have on immunotherapeutic options, is warranted. Due to their hyperproliferative state, cancer cells require a tremendous amount of energy and hence leave their TME in a state of chronic glucose and nutrient deprivation [71]. In effect, this glucose depleted condition renders immunotherapy inefficient as several immune cells, including T cells, natural killer (NK) cells, tumor associated macrophages (TAMs), and dendritic cells (DCs) utilize glucose for sustenance [72,73,74]. In addition to ATP generation, mitochondrial OXPHOS is a major cellular source of ROS, mainly H_2_O_2_ from complex I, II and III [75]. H_2_O_2_ has been reported to induce the phenotypic switch of macrophages and fibroblasts into a pro-inflammatory cancer-associated phenotype that further supports the multistep cascade of tumor progression and metastasis [76,77]. In addition, ROS play a significant role in lipid peroxidation that in turn, instigates pro-inflammatory immunosuppressive macrophages [78]. TAMs are also affected by reduced precursors to energy; lack of metabolites leads to increased polarization of protumorigenic M2 macrophages which are highly dependent on OxPhos and FAO [79]. In addition, the high extracellular lactate characteristic of the TME was cause a phenotypic switch from the pro-inflammatory M1 macrophage to the more immunosuppressive M2 macrophage [80]. Additionally, the influence of lactate on HIF expression, enhances the levels of arginase I and iNOS in M2-polarized macrophages [81], resulting in immunosuppressive T cell behavior. 

High levels of ROS in the TME inhibit T-cell proliferation and antitumor function leading to T-cell hyporesponsiveness. Tumor-infiltrating lymphocytes (TILs) suffer from reduced glycolytic capacity and cytokine production due to reduced Ca^2+^ signaling attributed to such a glucose deprived TME [82,83]. Low levels of ROS are required for T-cell activation, proliferation and function as T-cell activation through stimulation of the T cell receptors (TCR) and costimulatory receptors induce signaling pathways and transcription factors. TCR-dependent calcium influx into CD4^+^ T-cells leads to the generation of mitochondrial ROS from complex I and III of the ETC leading to CD4^+^ T-cell activation [84]. CD8 TILs exhibit functional and metabolic impairment, with high levels of mitochondrial ROS and downregulation of mitochondrial superoxide dismutase 2 (SOD2). The functional impairment and the antitumor activity of CD8 TILs were rescued by mitochondrial ROS scavengers [85,86] as well as the expression of PGC1α, the key player in mitochondrial biogenesis [87]. Furthermore, both ROS and RNS inhibit T-cell infiltration into tumors by inactivating CCL2 by nitration [88], and induce T-cell tolerance through the impairment of responsiveness and binding of CD8^+^ T-cells to peptide–MHC complexes [89].

Myeloid-derived suppressor cells (MDSCs), a subset of heterogeneous myeloid cells of polymorphonuclear (PMN-MDSC) or monocytic (M-MDSC) origin, thrive in conditions with high ROS levels that maintain the immunosuppressive properties of MDSCs. Consistently, in the TME, MDSCs produce high levels of ROS as one of the major mechanisms that MDSCs use to suppress not only T cells but also NK cell responses and cytotoxicity in tumors [90,91]. Importantly, redox-signaling and oxidative stress responses in MDSCs are mainly regulated by HIF-1α and Nrf2, both of which are critical orchestrators of MDSC fate and function [90,91]. 

Natural killer (NK) cells, a subset of myeloid cells, plays a significant role in infection, hematopoietic stem cell transplantation, autoimmunity, and tumor immune surveillance by virtue of their ability to spontaneously kill “stressed” target cells without prior sensitization or MHC restriction. ROS/RNS could reduce the cytotoxicity of NK cells and reverse the suppression of immunity [92]. The activity of NK cells and their antitumor effect has been reported to be regulated by the tumor suppressor GSK-3β through inhibition of the ROS/eIF2B pathway suggesting ROS as a potential target for cancer therapy [93]. 

With respect to dendritic cells (DCs), contradictory effects of hypoxic TME with excessive ROS have been reported [94]. Despite the effect of hypoxia and ROS in the upregulation of a plethora of pro-inflammatory cytokines, both hypoxia and ROS stimulate migration of immature but prevent migration of mature—DCs resulting in an immunosuppressive TME. These lead to limited influx and maturation of immature DCs to the TME while the egress of activated DCs to lymphoid system is prevented, limiting the function of DCs in presenting tumor antigens to activate T lymphocytes, [94]. 

In addition to the aforementioned effects of excessive ROS, hypoxia and lactic acidosis that result from excessive tumor cell glycolysis and glucose-depletion, the depletion of other nutrients in the TME had a profound effect on immune cells infiltration and phenotype. For example, glutamine depletion in the TME may increase regulatory T cells (Treg), resulting in a more immunosuppressive environment [95]. CD8+ T cells and NK cells, demonstrated decreased function in conditions of glutamine, serine, or glycine depletion [92,96,97]. 

The consequence of tumor cell metabolism on immune cells also diminishes the effectiveness of immunotherapies, many of which rely on fully functional immune cells to execute their antitumorigenic effects. The efficacy of the new immune checkpoint inhibitors and chimeric antigen receptor T cells (CAR -T) for cancer treatment is highly dependent on T cell proliferative ability and effector functions [87,98]. Immune-checkpoint inhibition by blockade of programmed death-1 (PD-1) signaling decreased both mitochondrial H_2_O_2_ and total cellular ROS levels [99]. The latter study revealed that PD-1–driven increase in ROS was not only dependent on FAO, as evidenced by reversal by etomoxir, but also on inhibited T cell survival, an effect that was mitigated by antioxidants [99]. Moreover, the upregulation of prostaglandins, derived from prostaglandin E2 synthase and cyclooxygenase (COX)-1/2-mediated catabolism of arachidonic acid, can also suppress T-cell function, an effect that was mitigated by COX inhibitors potentiating immune checkpoint therapy and improving CD8^+^ T-cell responses [100].

The upregulation of amino acid-degrading enzymes in the TME suppresses T-cell function including indoleamine-2,3-dioxygenase (IDO) and tryptophan-2,3-dioxygenase (TDO), which degrade tryptophan, and arginase-1 and nitric oxide synthase (NOS), which degrade l-arginine [101,102]. IDO inhibitors have been demonstrated to enhance CAR T-cell efficacy, an effect that led to the evaluation of IDO inhibitors in clinical trials [89,103,104]. Arginase activity has also been demonstrated to inhibit the proliferation and cytotoxicity of CAR T-cells, whereas the degradation of l-arginine by the NOS pathway generates RNS which inhibit T cell tumor infiltration and activation [89,103]. 

## 8. Transcription Factors and Redox Homeostasis

### 8.1. Hypoxia-Inducible Factor-1 (HIF-1)

Hypoxia-inducible factor-1 is a component of heterodimeric transcription factor HIF-1 that belongs to HIFs family (HIF-1, HIF-2 and HIF-3). It plays a pivotal role in the adaptive regulation of cellular function in hypoxic conditions [105]. HIF-1 is a heterodimer, composed of an O_2_-regulated HIF-1α subunit and a constitutively-expressed HIF-1β subunit [106,107]. HIF-1α contains three hydroxylation sites: two prolyl residues in the O_2_-dependent degradation domain (ODDD), and one aspartyl residue in the C-terminal transcription activation domain (C-TAD) [108]. HIF-1α proline residues 402 and 564 (Pro-402 and/or Pro-564) are hydroxylated in well-oxygenated cells via prolyl hydroxylase enzymes (PHDs). The substrates of this reaction are α-ketoglutarate and O_2,_ and the byproducts are succinate and CO_2_ [108]. The von Hippel-Lindau (VHL) tumor suppressor protein, an E3-ubiquitin ligase, binds prolyl-hydroxylated HIF-1α, targeting it for proteasomal degradation [108]. In hypoxic conditions, O_2_ (substrate) deprivation and/or ROS generation by mitochondria, which may oxidize ferrous ions present in the catalytic domain of the hydroxylases, inhibit the asparaginyl and prolyl hydroxylation reactions [109,110,111]. This leads to limited substrate availability for prolyl- hydroxylase (PHD) enzyme, leaving HIF-1α in a nonhydroxylated, and hence stabilized form that cannot be degraded by E3 ligases. Consequently, stabilized HIF-1α undergoes nuclear translocation, hetero-dimerizes with the HIF-1β subunit, and binds to hypoxia responsive elements (HRE) in the promoters of target genes [108]. Interestingly, ROS-induced stabilization of HIF-1α was blocked by Vitamin C and antioxidants as *N*-acetyl cysteine (NAC) [112,113,114,115]. Consistently, in hypoxic conditions, cancer cells exhibit persistent oxidative stress with increased intracellular ROS due to mitochondrial complex III deregulation [116]. Mitochondrial ROS and NADPH oxidases play a predominant role in HIF-1 stabilization in hypoxic as well as nonhypoxic situations [110,116,117,118]. For example, hypoxia induced a redox-dependent stabilization of HIF-1α that was dependent on NADPH oxidase-driven ROS, Ca^2+^ and the mammalian target of rapamycin (mTOR) signaling [113]. Thus, the feedforward loop of the regulation of the expression and activity HIF1α and ROS initiates a redox adaptation response, thereby increasing the tolerance of cancer cells to oxidative stress, with upregulation of survival pathways (Figure 3) [113,119,120,121].

HIF-1α is activated in cancer cells by the loss of function of tumor suppressors (e.g., VHL or PTEN) and/or oncogene gain of function (constitutive growth factor /receptor activation [122,123,124]. Rapidly proliferating tumor cells experience chronic hypoxia and reduced O_2_ availability that, in turn, induce HIF-1α, which regulates the transcription of a plethora of gene encoding proteins involved in every aspect of cancer biology including carcinogenesis, cell transformation; cell proliferation, genome instability/DNA mutation, inflammation, glucose and energy metabolism, angiogenesis, autocrine growth factor signaling, invasion, metastasis, immune evasion, stemness, and resistance to chemo- and radiation therapy [105,122,125,126,127,128,129,130,131,132,133]. 

HIF-1α serves as major bioenergetics sensor in cancer cells in solid and hematologic malignancies [114,115,131]. HIF-1α enhances aerobic glycolysis through the upregulation of glucose transporters, glucose uptake and hexokinases with enhancement of glycolysis (Figure 3) [122,125]. This enhanced glucose uptake by hypoxic, metabolically active tumor areas is the basis of FDG-PET imaging of tumors [122,125]. HIF-1α is a potent inducer of VEGF expression and its expression correlates significantly with VEGF expression and microvessel density (MVD). Both HIF-1α and VEGF positively correlated with tumor stage and negatively correlated with patients survival [132,133]. HIF-1α confers resistance on cancer cells due to glycolytic inhibitor 2-deoxyglucose, as well as biguanides such as metformin [112,129,134,135].

### 8.2. Peroxisome Proliferator-Activated Receptor Gamma Coactivator 1 Alpha (PGC-1α) 

PGC-1α serves as a central hub for metabolic pathways that modulate mitochondrial biogenesis and oxidative metabolism. It was first found to cooperate with peroxisome proliferator-activated receptor-γ (PPARγ) transcription factor in adipose-rich tissue and was subsequently found to be hypermutated in several tumors [136,137,138]. Environmental and biological stimuli dictate alterations in the activity of PGC-1α [136,137,138]. The PGC-1 family members interact with and potentiate the activity of a plethora of transcription factors, including PPARs, nuclear respiratory factor-1/2 (NRF1/2), yin yang 1 (YY1), and estrogen-related receptors (ERRs), which control the expression of a large number of mitochondrial proteins β-ATP synthase, cytochrome c, cytochrome c oxidase subunits, transcription factor A mitochondrial (TFAM) [139], and transcription factor B1 M and B2 M (TFB1M, TFB2M) [139].

**Figure 4:**
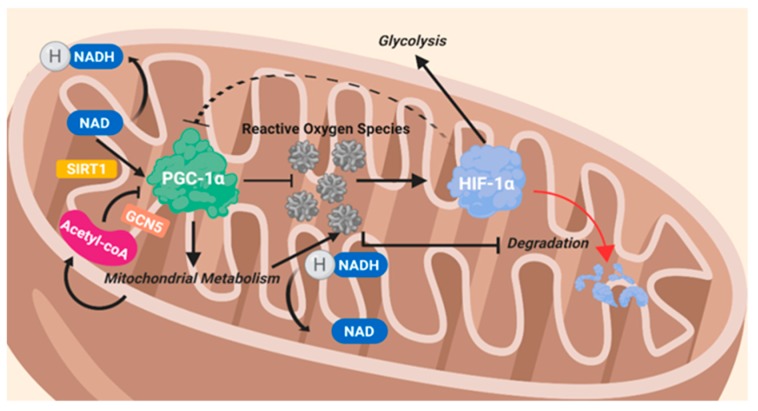
The intertwined reciprocal nature of PGC-1α and HIF-1α through ROS.

PGC-1α regulates the expression of the fatty acid transporters CD36, which allows fatty acids into the cell, and carnitine palmitoyltransferase I (CPT1), which grants fatty acids entrance into the mitochondria, where they are oxidized (Figure 4). Levels of ROS were found to be elevated following any increase in fatty acid oxidation via the ETC. PGC-1α instigates pyruvate dehydrogenase kinase 4 (PDK4), which prevents glucose oxidation by blocking PDH and thus enhancing glycogen synthesis [139]. PGC-1α plays a role in the production of specific ROS-destroying enzymes [139,140,141,142]. PGC-1α is known to be influenced by several signaling pathways which modify the energy status of cancer cells such as AMPK, mTORC1, HIF-1α, and glucose transporters [139].

Two different cellular subtypes have been reported in melanoma based on their PGC1α expression [143]. PGC-1α-high melanoma cells, exhibited an elevated rate of mitochondrial oxidative metabolism, along with ROS detoxification. This metabolic behavior made them OXPHOS-dependent and resilient to oxidative stress [143,144]. PGC-1α-high cells reflected a heightened proliferative index and cell survival however, their invasive properties were suppressed through PGC-1α- inhibitor of DNA binding 2 protein (ID2) and transcription factor 4 (TCF4) [144]. On the other hand, PGC-1α-low melanoma cells possess fewer mitochondria and are mainly glycolytic, making them susceptible to ROS-mediated apoptosis [143,144,145]. These cells exhibited a lower rate of proliferation while fortifying their ability to metastasize [143,144]. On another note, the induction of PGC-1α was found to play a role in chemoresistance by amplifying mitochondrial oxidative metabolism [146]. 

In breast cancer, PGC-1α was shown to stimulate nuclear receptors and transcription factors, including PPARα, estrogen-related receptor alpha (ERRα), NRF1, and NRF2, which in turn led to increased ATP production. While PGC-1α primarily influences mitochondrial respiration, other metabolic pathways, including glycolysis, glutaminolysis, fatty acid oxidation, and ROS detoxification [147], are also included in the litany of effectors. A study performed by LeBleu et al. [142], reported that breast cancer cells expressing high levels of PGC-1α exhibited an increase in mitochondrial metabolism, greatly magnifying their propensity to metastasize. In addition, PGC-1α increased the resistance of breast cancer cells to the biguanide, metformin [148]. Genetic and/or pharmacologic inhibition of PGC-1α suppresses ATP production, actin cytoskeleton remodeling, intravasation, extravasation, and cellular survival [142,148,149] and resensitizes breast cancer cells to standard of care therapies [150]. 

In pancreatic adenocarcinoma, *c-MYC* controls the activity of PGC-1α, by binding to its promoter and, hence, inhibiting its transcription. The c-*MYC/PGC-1α* ratio influences the metabolic phenotype of pancreatic adenocarcinoma cells [141]. c-MYC and PGC-1α are intertwined in an intricate balance that direct metabolic plasticity in pancreatic CSCs [141]. Differentiated pancreatic tumor cells highly express c-MYC and, hence, exhibit low levels of PGC-1α; however, in cancer stem cells (CSCs), c-MYC is not highly expressed and PGC-1α levels are amplified [141]. PGC-1α triggers the proliferation of androgen-dependent prostate cancer cells, through targeting AR-downstream target genes involved in cellular metabolism, mitochondrial biogenesis, as well as glucose and fatty acid oxidation [151]. PGC-1α is upregulated in prostate cancer cells through an androgen-AMPK feed-forward loop increasing mitochondrial metabolism [151]. PGC-1α-high prostate cancer cell xenografts in mice exhibited slower growth, progression and metastases [151]. Further studies indicated that this tumor suppressive characteristic of PGC-1α is facilitated by ERRα, yielding a transcriptionally catabolic outcome increasing β-oxidation and TCA cycle activity, diminishing the Warburg effect, and weakening tumor aggressiveness. Furthermore, the expression of PGC-1α negatively correlated with tumor grade, underscoring the prognostic value of PGC-1α in prostate cancer [140].

### 8.3. Nuclear Factor Erythroid 2-Related Factor 2 (NRF2) 

**Figure 5:**
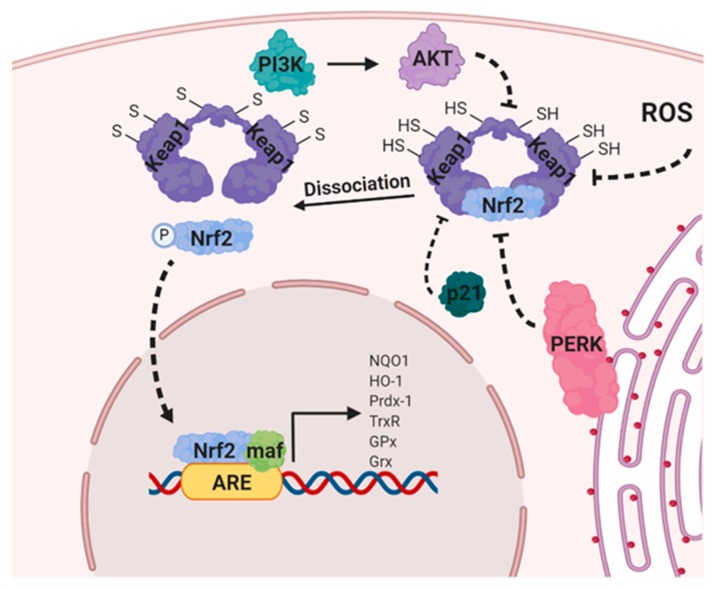
Inhibition of the Keap1/Nrf2 complex, leading to Nrf2 transcriptional activation.

Nrf2 is a transcription factor and the master regulator of cytoprotective reactions against oxidative stress [152], as it controls the transcription of a plethora of genes involved in the detoxification of ROS [153] (Figure 5). NRF2 is a basic leucine zipper (bZIP) transcription factor that regulates genes which contain antioxidant response elements (ARE) in their promoters [153]. Nrf2 is normally sequestered in the cytoplasm and is targeted for polyubiquitination and proteasomal degradation through its interaction with Kelch-like ECH-associated protein 1 (Keap1), a substrate/ binding partner of Cullin-3-(CUL3-) ring-box 1- (RBX1-) E3-ligase complex [152,153]. In the presence of ROS, key cystine residues on Keap1 are oxidized, leading to a conformational change which disrupts Keap1-Nrf2 interaction, allowing nuclear translocation of Nrf2 to occur, where it promotes transcription of target genes involved in response to injury and inflammation with the production of ROS [153]. NRF2 is also negatively regulated by two other E3 ubiquitin ligase complexes: the Beta-Transducin Repeat Containing E3 Ubiquitin Protein Ligase (β-TrCP)- S-Phase Kinase Associated Protein-1 (SKP1)-CUL1-RBX1 complex and HMG-CoA reductase degradation 1 homolog (HRD1, also known as Synoviolin 1) [153].

Cancer cells differ from normal cells by their enormous growth and proliferative capacity, which is often observed with Nrf2 overactivation. The reduced state of GSH is indispensable for cell proliferation due to its detoxification and antioxidant defense function. The excessive activation of Nrf2 greatly facilitates transcriptions of several genes involved in the formation of NADPH, the main cofactor in GSH synthesis [152]. Nrf2 overactivation is observed more often in cancer cells than in nontumorgenic cells, it results in the markedly elevated expression of G6PD, transketolase (TKT), phosphogluconate dehydrogenase (PGD), and other metabolic enzymes that lead to the metabolism of glucose and glutamine through the PPP and enhance the production of purine and amino acids, all of which lead to metabolic rewiring [152]. As several tumors depend on the NRF2-mediated cytoprotective response to counteract stress-induced conditions, targeting NRF2 pharmacologically can serve as a plausible and effective method to promote cancer cell death [152,153]. 

NRF2 can be activated by multiple upstream factors. Hyperactivation of the PI3K/AKT pathway activates NRF2 nuclear accumulation in renal adenocarcinoma cells [65,153]. Consistently, *KEAP1* or *NRF2* mutations in lung cancer and multiple myeloma cell lines result in the sustained activation of the PI3K/AKT pathway, coupled with increased *NRF2* transcript levels and nuclear localization. These responses allow enhanced metabolic reprogramming, cell proliferation, and apoptosis evasion [65,153] to occur. In addition, estrogen E2 enhances the expression of NRF2-dependent antioxidant genes in normal, malignant *BRCA1*-deficient cells as well as MCF-7 breast cancer cells, via the PI3K/ glycogen synthase kinase 3 beta (GSK3β) pathway [152]. Moreover, adenosine monophosphate kinase (AMPK), a highly established energy sensor phosphorylates NRF2 at the Ser550 position [154]. Furthermore, tumor suppressors *BRCA1* and p21 activate Nrf2 through the inhibition of the Keap1/Nrf2 complex [152,153]. Additionally, Nrf2 protects against inflammation-induced carcinogenesis. *Nrf2*-deficient mice exhibited increased carcinogen-induced stomach [155], liver [156], and bladder [157] cancers compared to their wild-type littermates. Several Nrf2 activators including resveratrol, and synthetic chemicals such as oltipraz and oleanane triterpenoids, influence NRF2 activity by modifying intermolecular disulfide bonds between two *KEAP1* molecules at Cys273 and Cys288, which serve to enhance NRF2 nuclear accumulation and hence, increase the transcription of Nrf2/ARE-regulated genes [152,153]. 

## 9. Targeting ROS through Metabolic Modulators for the Treatment of Cancer 

### 9.1. Orlistat

**Figure 6:**
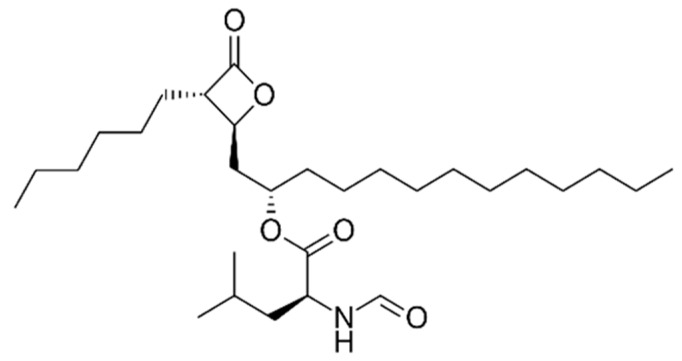
Structure of Orlistat.

Orlistat is a tetrahydrolipstatin (Figure 6) which is an FDA approved gastric and pancreatic lipase inhibitor that works in the intestine by hindering fat absorption by up to 30%, It is used mainly as a weight loss medication in obese patients [158]. Several studies investigated the effect of Orlistat as an antitumorigenic drug in various cancers such as T-cell Leukemia [158], melanoma [159], colorectal cancer [160,161], prostate cancer [162], hepatoma [163], breast cancer, and pancreatic cancer [164]. Orlistat suppresses tumor growth by inhibiting Fatty Acid Synthase (FASN), an anabolic multifunctional enzyme responsible for endogenous fatty acid synthesis from precursors acetyl-CoA and malonyl-CoA to make the 16 carbon polyunsaturated fatty acid palmitate [165,166]. It is a homodimer comprised of seven catalytic sites per polypeptide chain that act in a successive manner to produce palmitate. Fatty acids are essential for both tumorous and nontumorous cells as they play an essential role in energy storage and in signal transduction [159]. Thus, FASN is crucial for tumor cell survival, making it a desirable target for cancer therapeutics [158,165]. Orlistat has been found to irreversibly block the FASN thioesterase domain, the seventh functional domain, thus impeding its effect [163]. The thioesterase domain catalyzes the termination step by hydrolyzing the thioester bond between palmitate and the 4’-phosphopantetheine moiety of the acyl-carrier (ACP) domain [165,167]. Through its effects on FASN, Orlistat has been shown to decrease proliferation while stimulating apoptosis in various cancers [163,164]. Other studies have demonstrated that Orlistat inhibits cell growth and arrests the cell cycle at the G0/G1 phase [163]. In melanoma models, Orlistat not only decreased the cellular proliferation, and size of cancer xenografts in mice, and size and number of cervical and mediastinal lymph nodes, respectively, but also exerted an effect on the fatty acid composition of mitochondrial membranes, which were altered and served to upregulate apoptosis [159]. A study of the effect of Orlistat and NanoOrl, a nanoparticle formulation of Orlistat, on LNCaP and PC3 prostate, and on MDA-MB-231 breast cancer cell lines, showed that both formulations inhibited the thioesterase domain of FASN, and, therefore, lipid synthesis. Moreover, when combined with taxane drugs such as docetaxel, paclitaxel, and cabazitaxel, both Orlistat and NanoOrl showed robust synergy, and taxane resistance was overcome [166]. In another study, Chuang et al. found that Orlistat decreased NFκB, an upstream protein of FASN, and its downstream effector expressions in androgen-dependent and androgen independent cells (LNCaP and PC3 respectively). This effect, when combined with radiotherapy showed the greastest tumor suppression in both cell lines [162]. You et al. discovered that Orlistat inhibited cell growth of hepatoma Hep3B cells through FASN inhibition as well as through the induction of cell arrest at G0/G1. Furthermore, the study showed that the combination of both Orlistat and Paclitaxel had a strong synergistic effect on growth inhibition and cell apoptosis in Hep3B cells [163]. Additionally, when Orlistat was combined with Lonidamine and 6-Diazo-5-oxo-L-norleucine (DON) for treatment of SW480 colon cancer cells, they exhibited a synergistic cytotoxic effect with downregulation of their protein targets Hexokinase-2 (HK2), Glutaminase-1 (GLS-1), and Fatty Acid Synthase (FASN), respectively [160]. Finally, a study by Saleh et al. indicated that Orlistat exerted a cytotoxic effect and induced apoptosis in human breast cancer (MCF-7) and human pancreatic cancer (PANC-1) cell lines [164]. 

### 9.2. Biguandes (Metformin and Phenformin)

**Figure 7:**
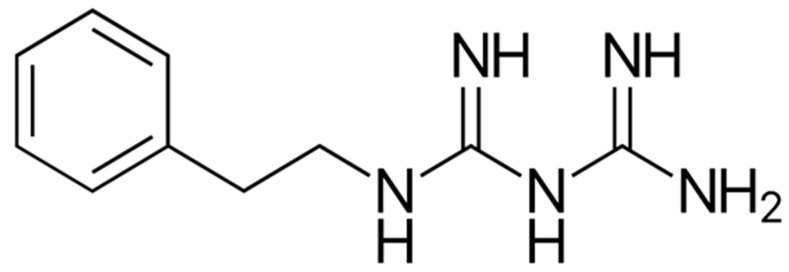
Structure of Phenformin.

Phenformin (Figure 7), is a member of the class of biguanides in which one of the terminal nitrogen atoms is substituted by a 2-phenylethyl group. It has been used in the past for treatment of type 2 diabetes mellitus [168]. However, it was later removed from the market due to its propensity to induce fatal lactic acidosis at high doses [168]. Remarkably, when combined with 2-DG, or dichloroacetate the lactic acidosis incidence was reduced [169]. While there are several proposed mechanisms of action, the main mechanism of action of biguanides is inhibiting complex I of the mitochondrial respiratory chain which causes an increase in the AMP/ATP ratio, hence activating AMPK [149,170,171]. In turn, AMPK is an invaluable energy sensor for cells that is activated in conditions of low ATP. In turn, AMPK downstream effectors suppress ATP anabolic processes and induce catabolic processes in an effort to increase ATP levels, leading to a decrease in protein and lipid synthesis, thereby, hindering tumor growth [172,173,174]. Phenformin can also reduce tumor cell progression through AMPK independent mechanisms [175,176]. The therapeutic effects of phenformin have been reported in melanoma [177] and breast cancer preclinical models, where it was shown to inhibit receptor tyrosine kinases as IGF1/IGF1R and ErbB2 with inhibition of mammary carcinogenesis, and cellular proliferation through cell cycle arrest at phase G0/G1 [178]. Moreover, phenformin significantly decreased the oxygen consumption in a dose and time dependent manner as detected by mitochondrial stress assays [179]. Another study indicated that malignant lymphocytes show an intrinsic resistance to biguanides such as phenformin and metformin that could be overcome by the disruption of the mitochondrial-derived ROS/HIF1-α axis, leading to the resensitization of malignant lymphocytes against phenformin [180]. With respect to bladder cancer, it was found that treatment with phenformin resulted in growth inhibition [181]. Bladder and ovarian cancer cell lines exposed to increasing concentrations of metformin and phenformin isomers of showed dramatic growth inhibition with more potent inhibitory effects of phenformin on proliferation and colony formation assays as well as a synergistic effect with the EGFR inhibitor gefitinib [182]. The proposed mechanism of inhibition of tumorigenic growth involved AMPK activation leading to the inhibition of the mTOR pathway with further downregulation of its downstream effectors, 4EP1 and p70S6K [183]. Lea et al. [181] demonstrated that phenformin inhibited the growth and glucose uptake of bladder and colon cancer cell lines through the inhibition of AKT and ERK1/2. Moreover, phenformin exhibited a synergistic effect when combined with pirarubicin [184]. In conclusion, the biguanide family, specifically phenformin, possesses several antitumorigenic properties, making them strong therapeutic candidates.

### 9.3. AICAR

**Figure 8:**
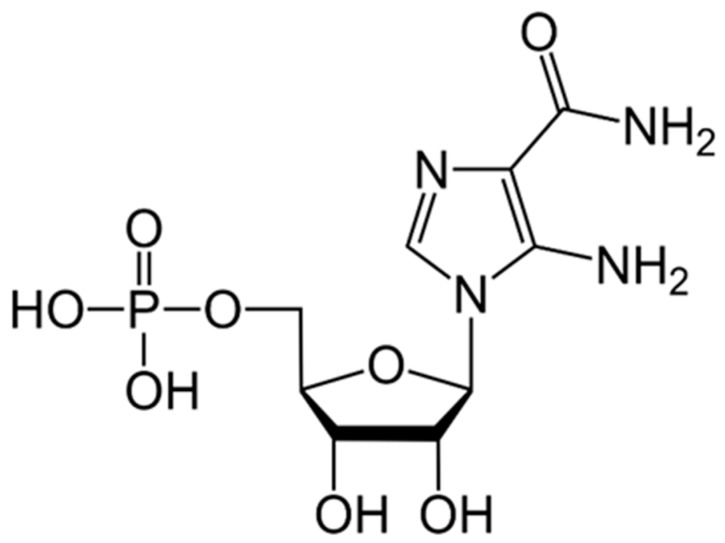
Structure of AICAR.

5-Aminoimidazole-4-carboxamide ribonucleoside or acadesine (AICAR) (Figure 8), an AMP analog, is a purine biosynthesis precursor and a recognized agonist of AMPK that was found to cause a myriad of widespread metabolic alterations in various tumors [185,186]. Mechanistically, AICAR enters the cell through adenosine transporters and becomes phosphorylated by adenosine kinase into 5-aminoimidazole-4-carboxamide ribonucleotide (AICAR) [186]. AICAR has been shown to have antitumorgenic properties in several types of cancer with tumor-selective antiproliferative and proapoptotic effects sparing normal cells [186,187,188]. These effects were mediated through direct inhibition of AKT-mTOR pathway in breast and cervical cancer cell lines [188,189]. Furthermore, AICAR inhibited prostate cancer proliferation and migration, induced apoptosis, and sensitized cells to chemo- and radiation therapy through AMPK/mTOR-dependent signaling pathway in both androgen-dependent and castration-resistant (CRPC) cell lines, with a more pronounced effect on CRPC cells [187,188]. In acute and chronic myeloid leukemia (AML and CML, respectively), AICAR inhibited cellular proliferation, with induction of cell cycle arrest in G1-phase, and apoptosis through activation of the DNA damage–associated enzyme checkpoint kinase 1 (Chk1) induced by pyrimidine depletion [186], the activation of AMPK, and the inhibition of mTOR pathway in BCR-ABL and Philadelphia-positive acute lymphoblastic leukemia [190,191]. These results suggest that a possible combination of AICAR and an inhibitor of mTOR, such as rapamycin, or other PI3K/AKT/mTOR pathway inhibitors might be beneficial in the treatment of childhood leukemias [192]. In bladder cancer, AICAR enhanced growth inhibition and cellular apoptosis induced by 10-hydroxycamptothecin in T24 and 5637 bladder cancer cell lines [193].

### 9.4. 2-Deoxyglucose (2DG)

**Figure 9:**
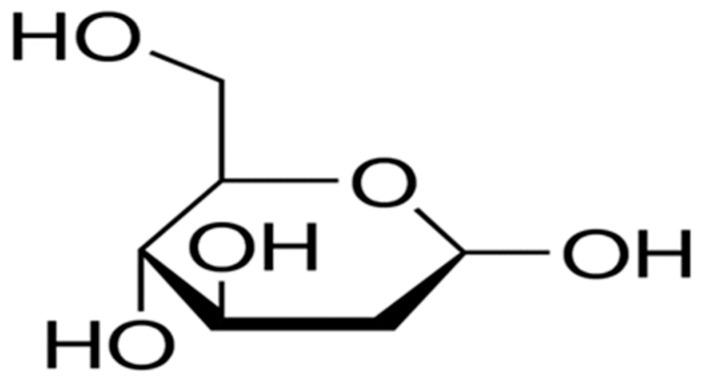
Structure of 2-DG.

2-Deoxyglucose (2DG, Figure 9), a nonmetabolizable glucose analog that is readily taken up by glucose transporters, it also serves as a competitive inhibitor of glucose for hexokinase activity as it is phosphorylated by 2-DG-6-phosphate inhibiting the rate limiting enzyme in glycolysis [194,195]. Basically, 2-DG has been shown to influence several cellular properties including bioenergetics, proliferation, oxidative stress, autophagy, and apoptosis when used in conjunction with the standard of care chemotherapeutic agents in solid and hematologic malignancies [196,197,198,199,200]. In vitro, 2-DG upregulated the expression of GLUT1 increasing its own uptake by breast cancer cells leading to the inhibition of cellular viability and clonogenic survival, while promoting apoptotic cell [201]. Also, 2-DG inhibited angiogenesis at concentrations that mainly affected endothelial cells but did not influence tumor cell viability [202]. In additions, 2-DG reduced cell viability and proliferation in mesothelioma cell lines and exerted a synergistic agent with cisplatin and pemetrexed; however, this combination had little effect on the size of the spheroids in 3D culture [199]. Moreover, 2-DG resensitized myriad of chemo- and radiotherapy-resistant cancer cells [203,204,205,206]. The effect of 2-DG involoves not only the inhibition of glycolysis as was previously believed, but also interferes with N-linked glycosylation [202,207,208,209]. Clinical trials showed the efficacy of oral 2DG as a single agent, and in combination with SOC chemo- and radiation therapy. A phase 1 clinical trial (NCT00096707) found that in patients with advanced solid tumors, 2DG alone or in combination with docetaxel revealed clinically tolerable effects [210]. In another phase I/II clinical trial, 2DG was combined with large fraction (5 Gy) radiotherapy in patients with human glioma; remarkable tolerance to the combination in all patients with no brain parenchyma damage occuring [211]. However, the use of 2DG in clinical trials was not successful due to insufficient dosage as 2DG should be used in amounts equal to or even exceeding glucose levels. Such doses were associated with hypoglycemia muscle weakness, and cardiotoxicity [210].

### 9.5. CPI-613

**Figure 10:**
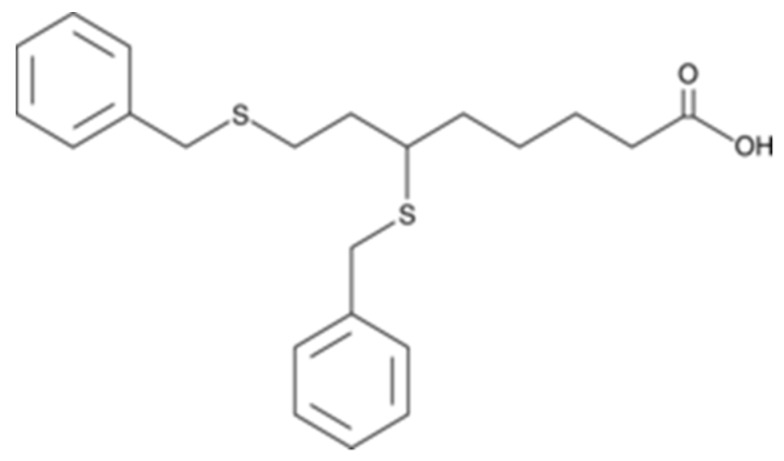
Structure of CPI-613.

CPI-613 also known as Devimistat is a lipoate analog (Figure 10) which inhibits pyruvate dehydrogenase (PDH) and α-ketoglutarate dehydrogenase complexes (KGDH), two essential enzymes in the tricarboxcylic acid (TCA) cycle [212]. TCA is the main route for OXPHOS in cancer cells; it orchestrates the metabolic and redox balance requirements [213]. CPI-613 is known to activate PDKs 1 to 4, leading to the inactivation of phosphorylation of PDH [214]. CPI-613 therefore triggers the activation of the E1α subunit of PDH, which inhibits cellular bioenergetics and instigates several mitochondrial pathways leading to tumor cell death. Moreover, CPI-613 generates an extensive, tumor-specific outflow of mitochondrial ROS, and hence inhibits mitochondrial metabolism [214,215]. CPI-613 re-sensitized acute myeloid leukemia (AML) cells to cytotoxic agents through inhibition of the TCA cycle, which is activated after exposure of AML cells to such agents [212]. While CPI-613 has several implications as a novel single agent, a phase I trial of CPI-613 in combination with cytarabine and mitoxantrone resulted in a complete remission in 50% of patients with recurrent AML. Moreover, in the combined Phase I/II study, elderly patients with recurrent AML who received CPI-613 had a significantly higher remission and median survival rates [212]. These inhibitory effects were also observed in H460 human nonsmall cell lung cancer cells and Saos-2 human sarcoma cells as well as in pancreatic tumors in in vivo xenograft models [215]. Additionally, it was shown that treatment with CPI-613 decreased the ability of ovarian cancer stem cells to form spheroids and hindered the enrichment of CD133^+^ and CD117^+^ stem cell population following olaparib and carboplatin/paclitaxel treatment without significantly affecting the survival of the nonstem cell population [216]. To further support the inhibitory effect of CPI-613, in a recent study [217], a unique copolymer was used to simultaneously deliver CPI-613 and LY2109761 (a TGF-β receptor I/II inhibitor) to stromal and cancerous cells. The authors reported significant inhibition of tumor growth by selectively incapacitating tumor and stromal cells [218].

### 9.6. Etomoxir

**Figure 11:**
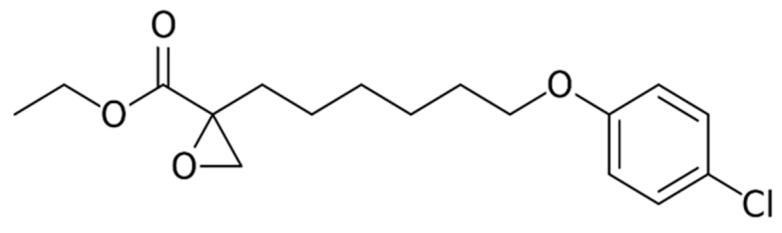
Structure of Etomoxir.

Etomoxir, or 2[6(4-chlorophenoxy) hexyl] oxirane-2-carboxylate **(**Figure 11**.)**, is classified as an irreversible inhibitor of carnitine palmitoyltransferase 1a (CPT1a), a transporter which is vital for the oxidation of mitochondrial long chain fatty acids [219]. Etomoxir also hinders complex I of the ETC [47]. With the administration of etomoxir, fatty acid influx into the mitochondria and β-oxidation decrease, with an increase in fatty acids in the cytosol as well as glucose oxidation [220]. The treatment of tumor-bearing mice with etomoxir significantly delayed tumor growth with less macrophage infiltration compared with untreated mice [221]. Moreover, the inhibition of fatty acid oxidation by etomoxir resulted in lipid accumulation, diminished ATP, and NADPH levels, and repressed bladder cancer cell growth both in vitro and in vivo [46]. This effect on bladder cancer cells was mediated through the PPARγ pathway and by altering fatty acid metabolism associated gene expression profiles leading to cell cycle arrest and inhibition of EMT that were reversed by PPARγ antagonist, GW9662 [46]. Interestingly, the dual targeting of resistant and highly aggressive cancer cells by 2DG and etomoxir inhibited cell proliferation and sensitized these cells to chemotherapy-induced apoptosis [43,222]. Etomoxir inhibited cell viability in glioblastoma SF188 cells with significant reduction of ATP, and NADPH levels [223]. Moreover, etomoxir induced oxidative stress and activated the proapoptotic LKB-1/AMPK pathway both of which can be attributed to the chemo-sensitizing capacity of the agent. Furthermore, etomoxir enhanced radiation efficacy against spheroids derived from lung and prostate cancers, eliminated hypoxic regions and significantly decreased proliferation (Ki-67 and cyclin D1), as well as stemness (CD44) and β-oxidation (CPT1A) markers compared to either etomoxir or radiotherapy alone [224]. 

## 10. Conclusion and Clinical Perspective:

**Figure 12:**
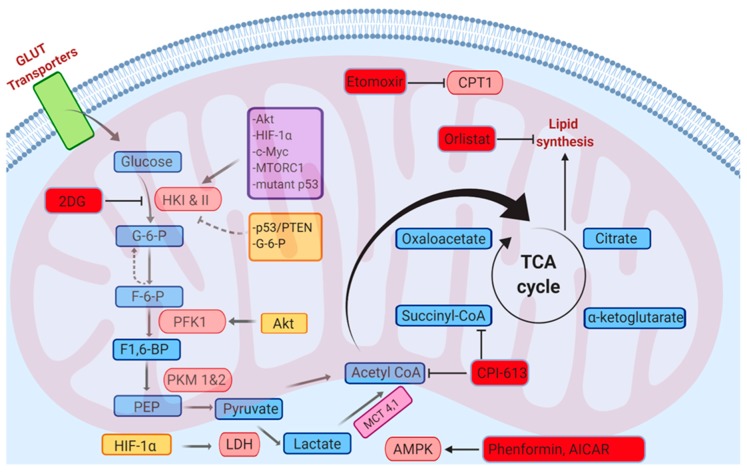
Effects of various metabolic modulators on the bioenergetics of cancer cells.

The translational potential of redox homeostasis has long revolved around manipulation of the redox balance. Because ROS can act as a double-edged sword with intricate balance to be found between generation and elimination, it is more likely to be advantageous to target the metabolic pathways that lead to the excessive ROS generation and/or the perturbed redox signaling pathways (Figure 12). 

## References

[1] Sullivan L., Chandel N.S. (2014). Mitochondrial reactive oxygen species and cancer. Cancer Metab..

[2] Brieger K., Schiavone S., Miller F.J., Krause K.-H. (2012). Reactive oxygen species: From health to disease. Swiss Med. Wkly..

[3] Parvez S., Long M.J.C., Poganik J.R., Aye Y. (2018). Redox Signaling by Reactive Electrophiles and Oxidants. Chem. Rev..

[4] Pizzino G., Irrera N., Bitto A., Pallio G., Mannino F., Arcoraci V., Aliquò F., Minutoli L., De Ponte C., D’Andrea P. (2017). Cadmium-Induced Oxidative Stress Impairs Glycemic Control in Adolescents. Oxidative Med. Cell. Longev..

[5] Niccoli S., Boreham D.R., Phenix C.P., Lees S.J. (2017). Non-radioactive 2-deoxy-2-fluoro-D-glucose inhibits glucose uptake in xenograft tumours and sensitizes HeLa cells to doxorubicin in vitro. PLoS ONE.

[6] Calvani M., Subbiani A., Vignoli M., Favre C. (2019). Spotlight on ROS and beta3-Adrenoreceptors Fighting in Cancer Cells. Oxid. Med. Cell Longev..

[7] Winterbourn C.C. (2008). Reconciling the chemistry and biology of reactive oxygen species. Nat. Methods.

[8] Winterbourn C.C. (2013). The Biological Chemistry of Hydrogen Peroxide. Methods Enzymol..

[9] Lancaster J.R. (2015). Nitric oxide: A brief overview of chemical and physical properties relevant to therapeutic applications. Futur. Sci. OA.

[10] Trachootham D., Lu W., Ogasawara M.A., Valle N.R.-D., Huang P. (2008). Redox Regulation of Cell Survival. Antioxidants Redox Signal..

[11] Gorrini C., Harris I., Mak T.W. (2013). Modulation of oxidative stress as an anticancer strategy. Nat. Rev. Drug Discov..

[12] Liu Y., Fiskum G., Schubert D. (2002). Generation of reactive oxygen species by the mitochondrial electron transport chain. J. Neurochem..

[13] Meitzler J.L., Antony S., Wu Y., Juhasz A., Liu H., Jiang G., Lu J., Roy K., Doroshow J.H. (2014). NADPH Oxidases: A Perspective on Reactive Oxygen Species Production in Tumor Biology. Antioxid. Redox Signal..

[14] Bogeski I., Niemeyer B.A. (2014). Redox Regulation of Ion Channels. Antioxid. Redox Signal..

[15] Agarwal A., Gupta S., Sekhon L., Shah R. (2008). Redox Considerations in Female Reproductive Function and Assisted Reproduction: From Molecular Mechanisms to Health Implications. Antioxid. Redox Signal..

[16] Agarwal A., Bui A.D. (2017). Oxidation-reduction potential as a new marker for oxidative stress: Correlation to male infertility. Investig. Clin. Urol..

[17] Lu J., Wang Z., Cao J., Chen Y., Dong Y. (2018). A novel and compact review on the role of oxidative stress in female reproduction. Reprod. Boil. Endocrinol..

[18] Wink D.A., Hines H.B., Cheng R., Switzer C.H., Flores-Santana W., Vitek M.P., Ridnour L.A., Colton C.A. (2011). Nitric oxide and redox mechanisms in the immune response. J. Leukoc. Boil..

[19] Nauseef W.M. (2007). How human neutrophils kill and degrade microbes: An integrated view. Immunol. Rev..

[20] Gostner J.M., Becker K., Fuchs D., Sucher R. (2013). Redox regulation of the immune response. Redox Rep..

[21] Lorenzen I., Mullen L., Bekeschus S., Hanschmann E.-M. (2017). Redox Regulation of Inflammatory Processes Is Enzymatically Controlled. Oxidative Med. Cell. Longev..

[22] Schatzman S., Culotta V.C. (2018). Chemical Warfare at the Microorganismal Level: A Closer Look at the Superoxide Dismutase Enzymes of Pathogens. ACS Infect. Dis..

[23] Diehn M., Cho R.W., Lobo N.A., Kalisky T., Dorie M.J., Kulp A.N., Qian D., Lam J.S., Ailles L.E., Wong M. (2009). Association of reactive oxygen species levels and radioresistance in cancer stem cells. Nature.

[24] Saha S.K., Bin Lee S., Won J., Choi H.Y., Kim K., Yang G.-M., Dayem A.A., Cho S.G. (2017). Correlation between Oxidative Stress, Nutrition, and Cancer Initiation. Int. J. Mol. Sci..

[25] Valko M., Rhodes C., Moncol J., Izakovic M., Mazur M. (2006). Free radicals, metals and antioxidants in oxidative stress-induced cancer. Chem. Interact..

[26] Valko M., Izakovic M., Mazúr M., Rhodes C.J., Telser J. (2004). Role of oxygen radicals in DNA damage and cancer incidence. Mol. Cell. Biochem..

[27] Sosa V., Moliné T., Somoza R., Paciucci R., Kondoh H., Lleonart M.E. (2013). Oxidative stress and cancer: An overview. Ageing Res. Rev..

[28] Chiang A.C., Massagué J. (2008). Molecular Basis of Metastasis. N. Engl. J. Med..

[29] Garrett R., Grisham C.M. (2013). Biochemistry.

[30] Weinhouse S., Warburg O., Burk D., Schade A.L. (1956). On Respiratory Impairment in Cancer Cells. Science.

[31] Warburg O. (1956). On the Origin of Cancer Cells. Science.

[32] Warburg O., Wind F., Negelein E. (1927). The Metabolism of Tumors in the Body. J. Gen. Physiol..

[33] Kim S.-Y. (2018). Cancer Energy Metabolism: Shutting Power off Cancer Factory. Biomol. Ther..

[34] Panieri E., Santoro M.M. (2016). ROS homeostasis and metabolism: A dangerous liason in cancer cells. Cell Death Dis..

[35] Cairns R.A., Harris I., Mak T.W. (2011). Regulation of cancer cell metabolism. Nat. Rev. Cancer.

[36] Kroemer G., Pouysségur J. (2008). Tumor Cell Metabolism: Cancer’s Achilles’ Heel. Cancer Cell.

[37] Carracedo A., Cantley L.C., Pandolfi P.P. (2013). Cancer metabolism: Fatty acid oxidation in the limelight. Nat. Rev. Cancer.

[38] Jeon S.-M., Hay N. (2012). The dark face of AMPK as an essential tumor promoter. Cell. Logist..

[39] Kuo C.-Y., Ann D.K. (2018). When fats commit crimes: Fatty acid metabolism, cancer stemness and therapeutic resistance. Cancer Commun..

[40] Zaugg K., Yao Y., Reilly P.T., Kannan K., Kiarash R., Mason J., Huang P., Sawyer S.K., Fuerth B., Faubert B. (2011). Carnitine palmitoyltransferase 1C promotes cell survival and tumor growth under conditions of metabolic stress. Genome Res..

[41] Shi J., Fu H., Jia Z., He K., Fu L., Wang W. (2016). High Expression of CPT1A Predicts Adverse Outcomes: A Potential Therapeutic Target for Acute Myeloid Leukemia. EBioMedicine.

[42] Samudio I., Konopleva M. (2015). Targeting leukemia’s "fatty tooth". Blood.

[43] Samudio I., Harmancey R., Fiegl M., Kantarjian H., Konopleva M., Korchin B., Kaluarachchi K., Bornmann W., Duvvuri S., Taegtmeyer H. (2009). Pharmacologic inhibition of fatty acid oxidation sensitizes human leukemia cells to apoptosis induction. J. Clin. Investig..

[44] Ma Y., Wang W., Devarakonda T., Zhou H., Wang X.-Y., Salloum F.N., Spiegel S., Fang X. (2020). Functional analysis of molecular and pharmacological modulators of mitochondrial fatty acid oxidation. Sci. Rep..

[45] Senni N., Savall M., Cabrerizo Granados D., Alves-Guerra M.C., Sartor C., Lagoutte I., Gougelet A., Terris B., Gilgenkrantz H., Perret C. (2019). beta-catenin-activated hepatocellular carcinomas are addicted to fatty acids. Gut.

[46] Cheng S., Wang G., Wang Y., Cai L., Qian K., Ju L., Liu X., Xiao Y., Wang X. (2019). Fatty acid oxidation inhibitor etomoxir suppresses tumor progression and induces cell cycle arrest via PPARgamma-mediated pathway in bladder cancer. Clin. Sci. (Lond.).

[47] Yao C.H., Liu G.-Y., Wang R., Moon S.H., Gross R.W., Patti G.J. (2018). Identifying off-target effects of etomoxir reveals that carnitine palmitoyltransferase I is essential for cancer cell proliferation independent of beta-oxidation. PLoS Biol..

[48] Yajun C., Chen Y., Xiaosa L., Xiao W., Chen J., Wang Z., Bin X. (2017). Loss of Sun2 promotes the progression of prostate cancer by regulating fatty acid oxidation. Oncotarget.

[49] Patra K.C., Hay N. (2014). The pentose phosphate pathway and cancer. Trends Biochem. Sci..

[50] Riganti C., Gazzano E., Polimeni M., Aldieri E., Ghigo D. (2012). The pentose phosphate pathway: An antioxidant defense and a crossroad in tumor cell fate. Free. Radic. Boil. Med..

[51] D’Alessandro A., Cervia D., Catalani E., Gevi F., Zolla L., Casini G. (2014). Protective effects of the neuropeptides PACAP, substance P and the somatostatin analogue octreotide in retinal ischemia: A metabolomic analysis. Mol. BioSyst..

[52] Lucarelli G., Galleggiante V., Rutigliano M., Sanguedolce F., Cagiano S., Bufo P., Lastilla G., Maiorano E., Ribatti D., Giglio A. (2015). Metabolomic profile of glycolysis and the pentose phosphate pathway identifies the central role of glucose-6-phosphate dehydrogenase in clear cell-renal cell carcinoma. Oncotarget.

[53] Yin L., Kufe T., Avigan D., Kufe N. (2014). Targeting MUC1-C is synergistic with bortezomib in downregulating TIGAR and inducing ROS-mediated myeloma cell death. Blood.

[54] Yang L., Venneti S., Nagarath D. (2017). Glutaminolysis: A Hallmark of Cancer Metabolism. Annu. Rev. Biomed. Eng..

[55] Izaki S., Goto H., Yokota S. (2008). Increased chemosensitivity and elevated reactive oxygen species are mediated by glutathione reduction in glutamine deprived neuroblastoma cells. J. Cancer Res. Clin. Oncol..

[56] Xiang L., Mou J., Shao B., Wei Y., Liang H., Takano N., Semenza G.L., Xie G. (2019). Glutaminase 1 expression in colorectal cancer cells is induced by hypoxia and required for tumor growth, invasion, and metastatic colonization. Cell Death Dis..

[57] Seltzer M.J., Bennett B.D., Joshi A.D., Gao P., Thomas A.G., Ferraris D., Tsukamoto T., Rojas C.J., Slusher B.S., Rabinowitz J.D. (2010). Inhibition of glutaminase preferentially slows growth of glioma cells with mutant IDH1. Cancer Res..

[58] Emadi A., Jun S.A., Tsukamoto T., Fathi A.T., Minden M.D., Dang C.V. (2014). Inhibition of glutaminase selectively suppresses the growth of primary acute myeloid leukemia cells with IDH mutations. Exp. Hematol..

[59] Li T., Le A. (2018). Glutamine Metabolism in Cancer. Adv. Exp. Med. Biol..

[60] Vanhove K., Derveaux E., Graulus G.-J., Mesotten L., Thomeer M., Noben J.-P., Guedens W., Adriaensens P. (2019). Glutamine Addiction and Therapeutic Strategies in Lung Cancer. Int. J. Mol. Sci..

[61] Li L., Fath M.A., Scarbrough P.M., Watson W.H., Spitz D.R. (2014). Combined inhibition of glycolysis, the pentose cycle, and thioredoxin metabolism selectively increases cytotoxicity and oxidative stress in human breast and prostate cancer. Redox Boil..

[62] Locasale J.W. (2013). Serine, glycine and one-carbon units: Cancer metabolism in full circle. Nat. Rev. Cancer.

[63] Rosenzweig A., Blenis J., Gomes A.P. (2018). Beyond the Warburg Effect: How Do Cancer Cells Regulate One-Carbon Metabolism?. Front. Cell Dev. Boil..

[64] DeNicola M.G., Cantley L.C. (2015). Cancer’s Fuel Choice: New Flavors for a Picky Eater. Mol. Cell.

[65] DeNicola G.M., Chen P.-H., Mullarky E., Sudderth J.A., Hu Z., Wu D., Tang H., Xie Y., Asara J.M., Huffman K.E. (2015). NRF2 regulates serine biosynthesis in non–small cell lung cancer. Nat. Genet..

[66] Cheng Z., Ristow M. (2013). Mitochondria and Metabolic Homeostasis. Antioxid. Redox Signal..

[67] Ristow M. (2006). Oxidative metabolism in cancer growth. Curr. Opin. Clin. Nutr. Metab. Care.

[68] Wen X., Wu J., Wang F., Liu B., Huang C., Wei Y. (2013). Deconvoluting the role of reactive oxygen species and autophagy in human diseases. Free. Radic. Boil. Med..

[69] Marzetti E., Calvani R., Cesari M., Buford T.W., Lorenzi M., Behnke B.J., Leeuwenburgh C. (2013). Mitochondrial dysfunction and sarcopenia of aging: From signaling pathways to clinical trials. Int. J. Biochem. Cell Boil..

[70] Fath M.A., Diers A.R., Aykin-Burns N., Simons A.L., Hua L., Spitz D.R. (2009). Mitochondrial electron transport chain blockers enhance 2-deoxy-D-glucose induced oxidative stress and cell killing in human colon carcinoma cells. Cancer Boil. Ther..

[71] Badur M.G., Metallo C.M. (2017). Reverse engineering the cancer metabolic network using flux analysis to understand drivers of human disease. Metab. Eng..

[72] Chang C.-H., Qiu J., O’Sullivan D., Buck M., Noguchi T., Curtis J.D., Chen Q., Gindin M., Gubin M.M., Van Der Windt G.J. (2015). Metabolic Competition in the Tumor Microenvironment Is a Driver of Cancer Progression. Cell.

[73] Keating S.E., Zaiatz-Bittencourt V., Loftus R.M., Keane C., Brennan K., Finlay D.K., Gardiner C.M. (2016). Metabolic Reprogramming Supports IFN-gamma Production by CD56bright NK Cells. J. Immunol..

[74] O’Neill L.A.J., Pearce E.J. (2016). Immunometabolism governs dendritic cell and macrophage function. J. Cell Boil..

[75] Liemburg-Apers D., Willems P.H., Koopman W.J., Grefte S. (2015). Interactions between mitochondrial reactive oxygen species and cellular glucose metabolism. Arch. Toxicol..

[76] Naczki C., John B., Patel C., Lafferty A., Ghoneum A., Afify H., White M., Davis A., Jin G., Kridel S.J. (2018). SPARC Inhibits Metabolic Plasticity in Ovarian Cancer. Cancers.

[77] Said N., Frierson H.F., Sanchez-Carbayo M., Brekken R.A., Theodorescu D. (2013). Loss of SPARC in bladder cancer enhances carcinogenesis and progression. J. Clin. Investig..

[78] Serbulea V., Deweese D., Leitinger N. (2017). The effect of oxidized phospholipids on phenotypic polarization and function of macrophages. Free. Radic. Boil. Med..

[79] Renner K., Singer K., Koehl G.E., Geissler E.K., Peter K., Siska P.J., Kreutz M. (2017). Metabolic Hallmarks of Tumor and Immune Cells in the Tumor Microenvironment. Front. Immunol..

[80] Colegio O.R., Chu N.-Q., Szabo A.L., Chu T., Rhebergen A.M., Jairam V., Cyrus N., Brokowski C.E., Eisenbarth S.C., Phillips G.M. (2014). Functional polarization of tumour-associated macrophages by tumour-derived lactic acid. Nature.

[81] Ohashi T., Akazawa T., Aoki M., Kuze B., Mizuta K., Ito Y., Inoue N. (2013). Dichloroacetate improves immune dysfunction caused by tumor-secreted lactic acid and increases antitumor immunoreactivity. Int. J. Cancer.

[82] Ho P.-C., Bihuniak J.D., MacIntyre A., Staron M., Liu X., Amezquita R., Tsui Y.-C., Cui G., Micevic G., Perales J.C. (2015). Phosphoenolpyruvate Is a Metabolic Checkpoint of Anti-tumor T Cell Responses. Cell.

[83] Freitas C.M.T., Johnson D.K., Weber K.S. (2018). T Cell Calcium Signaling Regulation by the Co-Receptor CD5. Int. J. Mol. Sci..

[84] Devadas S., Zaritskaya L., Rhee S.G., Oberley L., Williams M.S. (2002). Discrete generation of superoxide and hydrogen peroxide by T cell receptor stimulation: Selective regulation of mitogen-activated protein kinase activation and fas ligand expression. J. Exp. Med..

[85] Kamiński M.M., Roth D., Sass S., Sauer S.W., Krammer P.H., Gülow K. (2012). Manganese superoxide dismutase: A regulator of T cell activation-induced oxidative signaling and cell death. Biochim. et Biophys. Acta (BBA)-Bioenerg..

[86] Kamiński M.M., Sauer S.W., Kaminski M., Opp S., Ruppert T., Grigaravicius P., Grudnik P., Gröne H.-J., Krammer P.H., Gülow K. (2012). T cell Activation Is Driven by an ADP-Dependent Glucokinase Linking Enhanced Glycolysis with Mitochondrial Reactive Oxygen Species Generation. Cell Rep..

[87] Scharping N.E., Menk A.V., Moreci R.S., Whetstone R.D., Dadey R.E., Watkins S.C., Ferris R.L., Delgoffe G.M. (2016). The Tumor Microenvironment Represses T Cell Mitochondrial Biogenesis to Drive Intratumoral T Cell Metabolic Insufficiency and Dysfunction. Immunity.

[88] Molon B., Ugel S., Del Pozzo F., Soldani C., Zilio S., Avella D., De Palma A., Mauri P., Monegal A., Rescigno M. (2011). Chemokine nitration prevents intratumoral infiltration of antigen-specific T cells. J. Exp. Med..

[89] Kosti P., Maher J., Arnold J. (2018). Perspectives on Chimeric Antigen Receptor T-Cell Immunotherapy for Solid Tumors. Front. Immunol..

[90] Ohl K., Fragoulis A., Klemm P., Baumeister J., Klock W., Verjans E., Böll S., Möllmann J., Lehrke M., Costa I. (2018). Nrf2 Is a Central Regulator of Metabolic Reprogramming of Myeloid-Derived Suppressor Cells in Steady State and Sepsis. Front. Immunol..

[91] Ohl K., Tenbrock K. (2018). Reactive Oxygen Species as Regulators of MDSC-Mediated Immune Suppression. Front. Immunol..

[92] Terrén I., Orrantia A., Vitallé J., Zenarruzabeitia O., Borrego F. (2019). NK Cell Metabolism and Tumor Microenvironment. Front. Immunol..

[93] Jin F., Wu Z., Hu X., Zhang J., Gao Z., Han X., Qin J., Li C., Wang Y. (2019). The PI3K/Akt/GSK-3beta/ROS/eIF2B pathway promotes breast cancer growth and metastasis via suppression of NK cell cytotoxicity and tumor cell susceptibility. Cancer Biol. Med..

[94] Paardekooper L., Vos W., Bogaart G.V.D. (2019). Oxygen in the tumor microenvironment: Effects on dendritic cell function. Oncotarget.

[95] Klysz D., Tai X., Robert P.A., Craveiro M., Cretenet G., Oburoglu L., Mongellaz C., Floess S., Fritz V., Matias M.I. (2015). Glutamine-dependent α-ketoglutarate production regulates the balance between T helper 1 cell and regulatory T cell generation. Sci. Signal..

[96] Ma E.H., Bantug G., Griss T., Condotta S., Johnson R.M., Samborska B., Mainolfi N., Suri V., Guak H., Balmer M.L. (2017). Serine Is an Essential Metabolite for Effector T Cell Expansion. Cell Metab..

[97] Swamy M., Pathak S., Grzes K.M., Damerow S., Sinclair L.V., Van Aalten D.M.F., Cantrell D.A. (2016). Glucose and glutamine fuel protein O-GlcNAcylation to control T cell self-renewal and malignancy. Nat. Immunol..

[98] Scharping N.E., Delgoffe G.M. (2016). Tumor Microenvironment Metabolism: A New Checkpoint for Anti-Tumor Immunity. Vaccines.

[99] Tkachev V., Goodell S., Opipari A.W., Hao L.-Y., Franchi L., Glick G.D., Ferrara J.L.M., Byersdorfer C. (2015). Programmed death-1 controls T cell survival by regulating oxidative metabolism. J. Immunol..

[100] Zelenay S., Van Der Veen A.G., Böttcher J.P., Snelgrove K.J., Rogers N., Acton S.E., Chakravarty P., Girotti M.R., Marais R., Quezada S.A. (2015). Cyclooxygenase-Dependent Tumor Growth through Evasion of Immunity. Cell.

[101] Munn D.H., Mellor A.L. (2007). Indoleamine 2,3-dioxygenase and tumor-induced tolerance. J. Clin. Investig..

[102] Munn D.H., Mellor A.L. (2016). IDO in the Tumor Microenvironment: Inflammation, Counter-Regulation, and Tolerance. Trends Immunol..

[103] Cheng J., Zhao L., Zhang Y., Qin Y., Guan Y., Zhang T., Liu C., Zhou J. (2019). Understanding the Mechanisms of Resistance to CAR T-Cell Therapy in Malignancies. Front. Oncol..

[104] Ninomiya S., Narala N., Huye L., Yagyu S., Savoldo B., Dotti G., Heslop H.E., Brenner M.K., Rooney C.M., Ramos C.A. (2015). Tumor indoleamine 2,3-dioxygenase (IDO) inhibits CD19-CAR T cells and is downregulated by lymphodepleting drugs. Blood.

[105] Masoud G.N., Li W. (2015). HIF-1α pathway: Role, regulation and intervention for cancer therapy. Acta Pharm. Sin. B.

[106] Wang G.L., Jiang B.-H., Rue E.A., Semenza G.L. (1995). Hypoxia-inducible factor 1 is a basic-helix-loop-helix-PAS heterodimer regulated by cellular O2 tension. Proc. Natl. Acad. Sci. USA.

[107] Wang G.L., Semenza G.L. (1995). Purification and Characterization of Hypoxia-inducible Factor 1. J. Boil. Chem..

[108] Kaelin W.G. (2017). The VHL Tumor Suppressor Gene: Insights into Oxygen Sensing and Cancer. Trans. Am. Clin. Clim. Assoc..

[109] Guzy R.D., Hoyos B., Robin E., Chen H., Liu L., Mansfield K.D., Simon M.C., Hämmerling U., Schumacker P.T. (2005). Mitochondrial complex III is required for hypoxia-induced ROS production and cellular oxygen sensing. Cell Metab..

[110] Guzy R.D., Schumacker P.T. (2006). Oxygen sensing by mitochondria at complex III: The paradox of increased reactive oxygen species during hypoxia. Exp. Physiol..

[111] Guzy R.D., Mack M.M., Schumacker P.T. (2007). Mitochondrial Complex III is Required for Hypoxia-Induced ROS Production and Gene Transcription in Yeast. Antioxid. Redox Signal..

[112] Gao P., Zhang H., Dinavahi R., Li F., Xiang Y., Raman V., Bhujwalla Z.M., Felsher D.W., Cheng L., Pevsner J. (2007). HIF-Dependent Antitumorigenic Effect of Antioxidants In Vivo. Cancer Cell.

[113] Calvani M., Comito G., Giannoni E., Chiarugi P. (2012). Time-Dependent Stabilization of Hypoxia Inducible Factor-1α by Different Intracellular Sources of Reactive Oxygen Species. PLoS ONE.

[114] Calvani M., Trisciuoglio D., Bergamaschi C., Shoemaker R.H., Melillo G. (2008). Differential Involvement of Vascular Endothelial Growth Factor in the Survival of Hypoxic Colon Cancer Cells. Cancer Res..

[115] Calvani M., Rapisarda A., Uranchimeg B., Shoemaker R.H., Melillo G. (2006). Hypoxic induction of an HIF-1α–dependent bFGF autocrine loop drives angiogenesis in human endothelial cells. Blood.

[116] Comito G., Calvani M., Giannoni E., Bianchini F., Calorini L., Torre E., Migliore C., Giordano S., Chiarugi P. (2011). HIF-1α stabilization by mitochondrial ROS promotes Met-dependent invasive growth and vasculogenic mimicry in melanoma cells. Free. Radic. Boil. Med..

[117] Patten D.A., LaFleur V.N., Robitaille G.A., Chan D.A., Giaccia A.J., Richard D.E. (2010). Hypoxia-inducible Factor-1 Activation in Nonhypoxic Conditions: The Essential Role of Mitochondrial-derived Reactive Oxygen Species. Mol. Boil. Cell.

[118] Lee H.-Y., Lee T., Lee N., Yang E.G., Lee C., Lee J., Moon E.-Y., Ha J., Park H. (2011). Src activates HIF-1α not through direct phosphorylation of HIF-1α-specific prolyl-4 hydroxylase 2 but through activation of the NADPH oxidase/Rac pathway. Carcinogenesis.

[119] Kotake-Nara E., Saida K. (2007). Characterization of CoCl2-induced reactive oxygen species (ROS): Inductions of neurite outgrowth and endothelin-2/vasoactive intestinal contractor in PC12 cells by CoCl2 are ROS dependent, but those by MnCl2 are not. Neurosci. Lett..

[120] Kotake-Nara E., Saida K. (2006). Endothelin-2/Vasoactive Intestinal Contractor: Regulation of Expression via Reactive Oxygen Species Induced by CoCl22, and Biological Activities Including Neurite Outgrowth in PC12 Cells. Sci. World J..

[121] Kotake-Nara E., Takizawa S., Quan J., Wang H., Saida K. (2005). Cobalt chloride induces neurite outgrowth in rat pheochromocytoma PC-12 cells through regulation of endothelin-2/vasoactive intestinal contractor. J. Neurosci. Res..

[122] Semenza G.L. (2013). HIF-1 mediates metabolic responses to intratumoral hypoxia and oncogenic mutations. J. Clin. Investig..

[123] Semenza G.L. (2007). HIF-1 mediates the Warburg effect in clear cell renal carcinoma. J. Bioenerg. Biomembr..

[124] Semenza G.L. (2007). Hypoxia-Inducible Factor 1 (HIF-1) Pathway. Sci. STKE.

[125] Semenza G.L. (2009). HIF-1: Upstream and downstream of cancer metabolism. Curr. Opin. Genet. Dev..

[126] Fridman I.A., Ponomarenko E.A., Makarova O.V., Postovalova E.A., Zolotova N.A., Khochanskiy D.N., Mkhitarov V.A., Tsvetkov I.S., Kosyreva A.M. (2020). Morphological Characteristic of Melanoma B16 Progression in C57BL/6 Mice with High and Low Resistance to Hypoxia. Bull. Exp. Boil. Med..

[127] Vaupel P., Multhoff G. (2020). Fatal Alliance of Hypoxia-/HIF-1alpha-Driven Microenvironmental Traits Promoting Cancer Progression. Adv. Exp. Med. Biol..

[128] Tirpe A.A., Gulei D., Ciortea S.M., Crivii C., Berindan-Neagoe I. (2019). Hypoxia: Overview on Hypoxia-Mediated Mechanisms with a Focus on the Role of HIF Genes. Int. J. Mol. Sci..

[129] Nagao A., Kobayashi M., Koyasu S., Chow C., Harada H. (2019). HIF-1-Dependent Reprogramming of Glucose Metabolic Pathway of Cancer Cells and Its Therapeutic Significance. Int. J. Mol. Sci..

[130] Al Tameemi W., Dale T.P., Al-Jumaily R.M.K., Forsyth N.R. (2019). Hypoxia-Modified Cancer Cell Metabolism. Front. Cell Dev. Boil..

[131] Meng L., Cheng Y., Tong X., Gan S., Ding Y., Zhang Y., Wang C., Xu L., Zhu Y., Wu J. (2018). Tumor Oxygenation and Hypoxia Inducible Factor-1 Functional Inhibition via a Reactive Oxygen Species Responsive Nanoplatform for Enhancing Radiation Therapy and Abscopal Effects. ACS Nano.

[132] Shao J.-B., Li Z., Zhang N., Yang F., Gao W., Sun Z.-G. (2019). Hypoxia-inducible factor 1α in combination with vascular endothelial growth factor could predict the prognosis of postoperative patients with oesophageal squamous cell cancer. Pol. J. Pathol..

[133] Choi K.-S., Bae M.-K., Jeong J.-W., Moon H.-E., Kim K.-W. (2003). Hypoxia-induced angiogenesis during carcinogenesis. J. Biochem. Mol. Biol..

[134] Brahimi-Horn M.C., Pouysségur J. (2007). Harnessing the hypoxia-inducible factor in cancer and ischemic disease. Biochem. Pharmacol..

[135] Shaw R.J. (2006). Glucose metabolism and cancer. Curr. Opin. Cell Boil..

[136] Puigserver P. (2005). Tissue-specific regulation of metabolic pathways through the transcriptional coactivator PGC1-α. Int. J. Obes..

[137] Puigserver P., Rhee J., Lin J., Wu Z., Yoon J.C., Zhang C.Y., Krauss S., Mootha V.K., Lowell B.B., Spiegelman B.M. (2001). Cytokine stimulation of energy expenditure through p38 MAP kinase activation of PPARgamma coactivator-1. Mol. Cell.

[138] Puigserver P., Spiegelman B.M. (2003). Peroxisome Proliferator-Activated Receptor-γ Coactivator 1α (PGC-1α): Transcriptional Coactivator and Metabolic Regulator. Endocr. Rev..

[139] Bost F., Kaminski L. (2019). The metabolic modulator PGC-1α in cancer. Am. J. Cancer Res..

[140] Torrano V., Valcarcel L., Cortazar A.R., Liu X., Urosevic J., Castillo-Martin M., Fernández-Ruiz S., Morciano G., Caro-Maldonado A., Guiu M. (2016). The metabolic co-regulator PGC1α suppresses prostate cancer metastasis. Nature.

[141] Sancho P., Burgos-Ramos E., Tavera A., Bou Kheir T., Jagust P., Schoenhals M., Barneda D., Sellers K., Campos-Olivas R., Graña O. (2015). MYC/PGC-1α Balance Determines the Metabolic Phenotype and Plasticity of Pancreatic Cancer Stem Cells. Cell Metab..

[142] LeBleu V.S., O’Connell J.T., Herrera K.N.G., Wikman H., Pantel K., Haigis M.C., De Carvalho F.M., Damascena A., Chinen L., Rocha R.M. (2014). PGC-1α mediates mitochondrial biogenesis and oxidative phosphorylation in cancer cells to promote metastasis. Nature.

[143] Vazquez F., Lim J.-H., Chim H., Bhalla K., Girnun G., Pierce K., Clish C., Granter S.R., Widlund H.R., Spiegelman B.M. (2013). PGC1α expression defines a subset of human melanoma tumors with increased mitochondrial capacity and resistance to oxidative stress. Cancer Cell.

[144] Luo C., Widlund H.R., Puigserver P. (2016). PGC-1 Coactivators: Shepherding the Mitochondrial Biogenesis of Tumors. Trends Cancer.

[145] Piskounova E., Agathocleous M., Murphy M.M., Hu Z., Huddlestun S.E., Zhao Z., Leitch A.M., Johnson T.M., DeBerardinis R.J., Morrison S.J. (2015). Oxidative stress inhibits distant metastasis by human melanoma cells. Nature.

[146] Haq R., Shoag J., Andreu-Pérez P., Yokoyama S., Edelman H., Rowe G.C., Frederick D.T., Hurley A.D., Nellore A., Kung A. (2013). Oncogenic BRAF regulates oxidative metabolism via PGC1α and MITF. Cancer Cell.

[147] Vega R.B., Huss J.M., Kelly D.P. (2000). The Coactivator PGC-1 Cooperates with Peroxisome Proliferator-Activated Receptor α in Transcriptional Control of Nuclear Genes Encoding Mitochondrial Fatty Acid Oxidation Enzymes. Mol. Cell. Boil..

[148] Andrzejewski S., Klimcakova E., Johnson R.M., Tabariès S., Annis M.G., McGuirk S., Northey J.J., Chenard V., Sriram U., Papadopoli D.J. (2017). PGC-1α Promotes Breast Cancer Metastasis and Confers Bioenergetic Flexibility against Metabolic Drugs. Cell Metab..

[149] Andrzejewski S., Siegel P.M., St-Pierre J. (2018). Metabolic Profiles Associated with Metformin Efficacy in Cancer. Front. Endocrinol..

[150] Deblois G., Smith H.W., Tam I.S., Gravel S.P., Caron M., Savage P., Labbé D.P., Bégin L.R., Tremblay M.L., Park M. (2016). ERRalpha mediates metabolic adaptations driving lapatinib resistance in breast cancer. Nat. Commun..

[151] Tennakoon J.B., Shi Y., Han J.J., Tsouko E., White M.A., Burns A.R., Zhang A., Xia X., Ilkayeva O.R., Xin L. (2013). Androgens regulate prostate cancer cell growth via an AMPK-PGC-1α-mediated metabolic switch. Oncogene.

[152] Jung B.-J., Yoo H.-S., Shin S., Park Y.-J., Jeon S.-M. (2018). Dysregulation of NRF2 in Cancer: From Molecular Mechanisms to Therapeutic Opportunities. Biomol. Ther..

[153] Leboeuf S.E., Wu W.L., Karakousi T.R., Karadal B., Jackson S.R., Davidson S.M., Wong K.-K., Koralov S.B., Sayin V.I., Papagiannakopoulos T. (2020). Activation of Oxidative Stress Response in Cancer Generates a Druggable Dependency on Exogenous Non-essential Amino Acids. Cell Metab..

[154] Joo M.S., Kim W.D., Lee K.Y., Kim J.H., Koo J.H., Kim S.G. (2016). AMPK Facilitates Nuclear Accumulation of Nrf2 by Phosphorylating at Serine 550. Mol. Cell. Boil..

[155] Ramos-Gomez M., Kwak M.K., Dolan P., Itoh K., Tamamoto M., Talalay P., Kensler T.W. (2001). Sensitivity to carcinogenesis is increased and chemoprotective efficacy of enzyme inducers is lost in *nrf2* transcription factor-deficient mice. Proc. Natl. Acad. Sci. USA.

[156] Xu D., Xu M., Jeong S., Qian Y., Wu H., Xia Q., Kong X. (2019). The Role of Nrf2 in Liver Disease: Novel Molecular Mechanisms and Therapeutic Approaches. Front. Pharmacol..

[157] Iida K. (2004). Nrf2 Is Essential for the Chemopreventive Efficacy of Oltipraz against Urinary Bladder Carcinogenesis. Cancer Res..

[158] Cioccoloni G., Aquino A., Notarnicola M., Caruso M.G., Bonmassar E., Zonfrillo M., Caporali S., Faraoni I., Villivà C., Fuggetta M.P. (2019). Fatty acid synthase inhibitor orlistat impairs cell growth and down-regulates PD-L1 expression of a human T-cell leukemia line. J. Chemother..

[159] De Almeida L.Y., Mariano F.S., Bastos D.C., Cavassani K.A., Raphelson J., Mariano V.S., Agostini M., Moreira F.S., Coletta R.D., Mattos-Graner R.O. (2019). The antimetastatic activity of orlistat is accompanied by an antitumoral immune response in mouse melanoma. Cancer Chemother. Pharmacol..

[160] Schcolnik-Cabrera A. (2019). A combination of inhibitors of glycolysis, glutaminolysis and de novo fatty acid synthesis decrease the expression of chemokines in human colon cancer cells. Oncol. Lett..

[161] Czumaj A., Zabielska J., Pakiet A., Mika A., Rostkowska O., Makarewicz W., Kobiela J., Sledzinski T., Stelmanska E. (2019). In Vivo Effectiveness of Orlistat in the Suppression of Human Colorectal Cancer Cell Proliferation. Anticancer. Res..

[162] Chuang H.Y., Lee Y.P., Lin W.C., Lin Y.H., Hwang J.J. (2019). Fatty Acid Inhibition Sensitizes Androgen-Dependent and -Independent Prostate Cancer to Radiotherapy via FASN/NF-kappaB Pathway. Sci. Rep..

[163] You B.-J., Chen L.-Y., Hsu P.-H., Sung P.-H., Hung Y.-C., Lee H.-Z. (2019). Orlistat Displays Antitumor Activity and Enhances the Efficacy of Paclitaxel in Human Hepatoma Hep3B Cells. Chem. Res. Toxicol..

[164] Saleh A., Elfayoumi H.M., Youns M., Barakat W. (2018). Rutin and orlistat produce antitumor effects via antioxidant and apoptotic actions. Naunyn-Schmiedeberg’s Arch. Pharmacol..

[165] Kridel S.J., Houghton P.J., Germain G.S., Harwood F.C., Schuetz J.D., Stewart C.F., Buchdunger E., Traxler P. (2004). Orlistat Is a Novel Inhibitor of Fatty Acid Synthase with Antitumor Activity. Cancer Res..

[166] Souchek J., Davis A.L., Hill T.K., Holmes M.B., Qi B., Singh P.K., Kridel S.J., Mohs A.M. (2017). Combination Treatment with Orlistat-Containing Nanoparticles and Taxanes Is Synergistic and Enhances Microtubule Stability in Taxane-Resistant Prostate Cancer Cells. Mol. Cancer Ther..

[167] Pemble C.W., Johnson L.C., Kridel S.J., Lowther W.T. (2007). Crystal structure of the thioesterase domain of human fatty acid synthase inhibited by Orlistat. Nat. Struct. Mol. Boil..

[168] Yendapally R., Sikazwe D., Kim S.S., Ramsinghani S., Fraser-Spears R., Witte A.P., La-Viola B. (2020). A review of phenformin, metformin, and imeglimin. Drug Dev. Res..

[169] Kankotia S., Stacpoole P.W. (2014). Dichloroacetate and cancer: New home for an orphan drug?. Biochim. et Biophys. Acta (BBA)-Bioenerg..

[170] Rubiño M.E.G., Carrillo E., Alcalá G.R., Dominguez-Martin A., Marchal J.A., Tassi H.B., Martín D. (2019). Phenformin as an Anticancer Agent: Challenges and Prospects. Int. J. Mol. Sci..

[171] Pecinová A., Drahota Z., Kovalcikova J., Kovarova N., Pecina P., Alan L., Zima M., Houstek J., Mráček T. (2017). Pleiotropic Effects of Biguanides on Mitochondrial Reactive Oxygen Species Production. Oxidative Med. Cell. Longev..

[172] Hardie D.G. (2013). The LKB1-AMPK pathway-friend or foe in cancer?. Cancer Cell.

[173] Hardie D.G. (2013). AMPK: A Target for Drugs and Natural Products with Effects on Both Diabetes and Cancer. Diabetes.

[174] Hardie D.G., Ross F.A., Hawley S.A. (2012). AMP-activated protein kinase: A target for drugs both ancient and modern. Chem. Boil..

[175] Kalender A., Selvaraj A., Kim S.Y., Gulati P., Brûlé S., Viollet B., Kemp B.E., Bardeesy N., Dennis P., Schlager J.J. (2010). Metformin, Independent of AMPK, Inhibits mTORC1 in a Rag GTPase-Dependent Manner. Cell Metab..

[176] Vincent E.E., Coelho P.P., Blagih J., Griss T., Viollet B., Jones R.G. (2014). Differential effects of AMPK agonists on cell growth and metabolism. Oncogene.

[177] Petrachi T., Romagnani A., Albini A., Longo C., Argenziano G., Grisendi G., Dominici M., Ciarrocchi A., Dallaglio K. (2016). Therapeutic potential of the metabolic modulator phenformin in targeting the stem cell compartment in melanoma. Oncotarget.

[178] Guo Z., Zhao M., Howard E.W., Zhao Q., Parris A.B., Ma Z., Yang X. (2017). Phenformin inhibits growth and epithelial-mesenchymal transition of ErbB2-overexpressing breast cancer cells through targeting the IGF1R pathway. Oncotarget.

[179] Geoghegan F., Chadderton N., Farrar G.J., Zisterer D., Porter R. (2017). Direct effects of phenformin on metabolism/bioenergetics and viability of SH-SY5Y neuroblastoma cells. Oncol. Lett..

[180] Khan H., Anshu A., Prasad A., Roy S., Jeffery J., Kittipongdaja W., Yang D.T., Schieke S.M. (2019). Metabolic Rewiring in Response to Biguanides Is Mediated by mROS/HIF-1a in Malignant Lymphocytes. Cell Rep..

[181] Lea M.A., Kim H., Desbordes C. (2018). Effects of Biguanides on Growth and Glycolysis of Bladder and Colon Cancer Cells. Anticancer. Res..

[182] Huang Y., Zhou S., He C., Deng J., Tao T., Su Q., Darko K.O., Peng M., Yang X. (2018). Phenformin alone or combined with gefitinib inhibits bladder cancer via AMPK and EGFR pathways. Cancer Commun..

[183] Zhou S., Xu L., Cao M., Wang Z., Xiao D., Xu S., Deng J., Hu X., He C., Tao T. (2019). Anticancer properties of novel pyrazole-containing biguanide derivatives with activating the adenosine monophosphate-activated protein kinase signaling pathway. Arch. der Pharm..

[184] Peng M., Deng J., Zhou S., Xiao D., Long J., Zhang N., He C., Mo M., Yang X. (2019). Dual Inhibition of Pirarubicin-Induced AKT and ERK Activations by Phenformin Sensitively Suppresses Bladder Cancer Growth. Front. Pharmacol..

[185] Rae C., Mairs R.J. (2019). AMPK activation by AICAR sensitizes prostate cancer cells to radiotherapy. Oncotarget.

[186] Dembitz V., Tomic B., Kodvanj I., Simon J.A., Bedalov A., Visnjic D. (2019). The ribonucleoside AICAr induces differentiation of myeloid leukemia by activating the ATR/Chk1 via pyrimidine depletion. J. Boil. Chem..

[187] Su C.-C., Hsieh K.-L., Liu P.-L., Yeh H.-C., Huang S.-P., Fang S.-H., Cheng W.-C., Huang S., Chiu F.-Y., Lin I.-L. (2019). AICAR Induces Apoptosis and Inhibits Migration and Invasion in Prostate Cancer Cells Through an AMPK/mTOR-Dependent Pathway. Int. J. Mol. Sci..

[188] Mukhopadhyay S., Chatterjee A., Kogan D., Patel D., Foster D.A. (2015). 5-Aminoimidazole-4-carboxamide-1-beta-4-ribofuranoside (AICAR) enhances the efficacy of rapamycin in human cancer cells. Cell Cycle.

[189] Guan T.-J., Qin F.-J., Du J.-H., Geng L., Zhang Y., Li M. (2007). AICAR inhibits proliferation and induced S-phase arrest, and promotes apoptosis in CaSki cells. Acta Pharmacol. Sin..

[190] Vakana E., Altman J.K., Glaser H., Donato N.J., Platanias L.C. (2011). Antileukemic effects of AMPK activators on BCR-ABL–expressing cells. Blood.

[191] Platanias L.C., Vakana E. (2011). AMPK in BCR-ABL expressing leukemias. Regulatory effects and therapeutic implications. Oncotarget.

[192] Sengupta T.K., Leclerc G.M., Hsieh-Kinser T.T., Leclerc G.J., Singh I., Barredo J.C. (2007). Cytotoxic effect of 5-aminoimidazole-4-carboxamide-1-beta-4-ribofuranoside (AICAR) on childhood acute lymphoblastic leukemia (ALL) cells: Implication for targeted therapy. Mol. Cancer.

[193] Wang Y., Cao F., Wang Y., Yu J., Jia B.-L. (2019). Silencing of SAA1 inhibits palmitate- or high-fat diet induced insulin resistance through suppression of the NF-kappaB pathway. Mol. Med..

[194] Aft R., Zhang F. (2009). Chemosensitizing and cytotoxic effects of 2-deoxy-D-glucose on breast cancer cells. J. Cancer Res. Ther..

[195] Lin X., Zhang F., Bradbury C.M., Kaushal A., Li L., Spitz D.R., Aft R.L., Gius D. (2003). 2-Deoxy-D-glucose-induced cytotoxicity and radiosensitization in tumor cells is mediated via disruptions in thiol metabolism. Cancer Res..

[196] Bénéteau M., Zunino B., Jacquin M.A., Meynet O., Chiche J., Pradelli L.A., Marchetti S., Cornille A., Carles M., Ricci J.-E. (2012). Combination of glycolysis inhibition with chemotherapy results in an antitumor immune response. Proc. Natl. Acad. Sci. USA.

[197] Reyes R., Wani N., Ghoshal K., Jacob S.T., Motiwala T. (2016). Sorafenib and 2-Deoxyglucose Synergistically Inhibit Proliferation of Both Sorafenib-Sensitive and -Resistant HCC Cells by Inhibiting ATP Production. Gene Expr..

[198] Tomizawa M., Shinozaki F., Motoyoshi Y., Sugiyama T., Yamamoto S., Ishige N. (2016). 2-Deoxyglucose and sorafenib synergistically suppress the proliferation and motility of hepatocellular carcinoma cells. Oncol. Lett..

[199] Gerogianni I., Pitaraki E., Jagirdar R.M., Kouliou O., Giannakou L., Giannopoulos S., Papazoglou E., Hatzoglou C., Gourgoulianis K.I., Zarogiannis S.G. (2019). 2-Deoxy-glucose Enhances the Effect of Cisplatin and Pemetrexed in Reducing Malignant Pleural Mesothelioma Cell Proliferation But Not Spheroid Growth. Anticancer. Res..

[200] Zhang N., Li J., Wang F., Hu J., Wang S., Sun Y. (2014). 2-Deoxy-D-glucose targeting of glucose metabolism in cancer cells as a potential therapy. Cancer Lett..

[201] Aft R.L., Zhang F.W., Gius D. (2002). Evaluation of 2-deoxy-D-glucose as a chemotherapeutic agent: Mechanism of cell death. Br. J. Cancer.

[202] Merchan J.R., Kovacs K., Railsback J.W., Kurtoglu M., Jing Y., Piña Y., Gao N., Murray T.G., Lehrman M.A., Lampidis T.J. (2010). Antiangiogenic Activity of 2-Deoxy-D-Glucose. PLoS ONE.

[203] Islamian J.P., Aghaee F., Farajollahi A., Baradaran B., Fazel M. (2015). Combined Treatment with 2-Deoxy-D-Glucose and Doxorubicin Enhances the in Vitro Efficiency of Breast Cancer Radiotherapy. Asian Pac. J. Cancer Prev..

[204] Oladghaffari M., Monfared A.S., Farajollahi A., Baradaran B., Mohammadi M., Shanehbandi D., Abadi M.A.J., Islamian J.P. (2017). MLN4924 and 2DG Combined Treatment Enhances the Efficiency of Radiotherapy in the Breast Cancer Cells. Int. J. Radiat. Boil..

[205] Ben Sahra I., Laurent K., Giuliano S., Larbret F., Ponzio G., Gounon P., Le Marchand-Brustel Y., Giorgetti-Peraldi S., Cormont M., Bertolotto C. (2010). Targeting Cancer Cell Metabolism: The Combination of Metformin and 2-Deoxyglucose Induces p53-Dependent Apoptosis in Prostate Cancer Cells. Cancer Res..

[206] Ben Sahra I., Tanti J.-F., Bost F. (2010). The combination of metformin and 2 deoxyglucose inhibits autophagy and induces AMPK-dependent apoptosis in prostate cancer cells. Autophagy.

[207] Kurtoglu M., Lampidis T.J. (2009). From delocalized lipophilic cations to hypoxia: Blocking tumor cell mitochondrial function leads to therapeutic gain with glycolytic inhibitors. Mol. Nutr. Food Res..

[208] Kurtoglu M., Gao N., Shang J., Maher J.C., Lehrman M.A., Wangpaichitr M., Savaraj N., Lane A.N., Lampidis T.J. (2007). Under normoxia, 2-deoxy-D-glucose elicits cell death in select tumor types not by inhibition of glycolysis but by interfering with N-linked glycosylation. Mol. Cancer Ther..

[209] Kurtoglu M., Maher J.C., Lampidis T.J. (2007). Differential Toxic Mechanisms of 2-Deoxy-D-Glucose versus 2-Fluorodeoxy-D -Glucose in Hypoxic and Normoxic Tumor Cells. Antioxid. Redox Signal..

[210] Raez L.E., Papadopoulos K., Ricart A.D., Chiorean E.G., DiPaola R.S., Stein M.N., Lima C.M.R., Schlesselman J.J., Tolba K., Langmuir V.K. (2012). A phase I dose-escalation trial of 2-deoxy-d-glucose alone or combined with docetaxel in patients with advanced solid tumors. Cancer Chemother. Pharmacol..

[211] Mohanti B.K., Rath G.K., Anantha N., Kannan V., Das B.S., Chandramouli B.A., Banerjee A.K., Das S., Jena A., Ravichandran R. (1996). Improving cancer radiotherapy with 2-deoxy-d-glucose: Phase I/II clinical trials on human cerebral gliomas. Int. J. Radiat. Oncol..

[212] Pardee T.S., Luther S., Buyse M., Powell B.L., Cortes J. (2019). Devimistat in combination with high dose cytarabine and mitoxantrone compared with high dose cytarabine and mitoxantrone in older patients with relapsed/refractory acute myeloid leukemia: ARMADA 2000 Phase III study. Futur. Oncol..

[213] Pardee T.S., Anderson R.G., Pladna K.M., Isom S., Ghiraldeli L.P., Miller L., Chou J.A., Jin G., Zhang W., Ellis L.R. (2018). A Phase I Study of CPI-613 in Combination with High-Dose Cytarabine and Mitoxantrone for Relapsed or Refractory Acute Myeloid Leukemia. Clin. Cancer Res..

[214] Stuart S.D., Schauble A., Gupta S., Kennedy A.D., Keppler B.K., Bingham P.M., Zachar Z. (2014). A strategically designed small molecule attacks alpha-ketoglutarate dehydrogenase in tumor cells through a redox process. Cancer Metab..

[215] Zachar Z., Marecek J., Maturo C., Gupta S., Stuart S.D., Howell K., Schauble A., Lem J., Piramzadian A., Karnik S. (2011). Non-redox-active lipoate derivates disrupt cancer cell mitochondrial metabolism and are potent anticancer agents in vivo. J. Mol. Med..

[216] Bellio C., DiGloria C., Spriggs D.R., Foster R., Growdon W.B., Rueda B.R. (2019). The Metabolic Inhibitor CPI-613 Negates Treatment Enrichment of Ovarian Cancer Stem Cells. Cancers.

[217] Lee N., Jang W.-J., Seo J.H., Lee S., Jeong C.-H. (2019). 2-Deoxy-d-Glucose-Induced Metabolic Alteration in Human Oral Squamous SCC15 Cells: Involvement of N-Glycosylation of Axl and Met. Metab..

[218] Li Y., Zhao Z., Liu H., Fetse J.P., Jain A., Lin C.-Y., Cheng K. (2019). Development of a Tumor-Responsive Nanopolyplex Targeting Pancreatic Cancer Cells and Stroma. ACS Appl. Mater. Interfaces.

[219] O’Connor R.S., Guo L., Ghassemi S., Snyder N.W., Worth A.J., Weng L., Kam Y., Philipson B., Trefely S., Nunez-Cruz S. (2018). The CPT1a inhibitor, etomoxir induces severe oxidative stress at commonly used concentrations. Sci. Rep..

[220] Xu F.Y., Taylor W.A., Hurd J.A., Hatch G.M. (2002). Etomoxir mediates differential metabolic channeling of fatty acid and glycerol precursors into cardiolipin in H9c2 cells. J. Lipid Res..

[221] Hossain F., Al-Khami A.A., Wyczechowska D., Hernandez C., Zheng L., Reiss K., Del Valle L., Trillo-Tinoco J., Maj T., Zou W. (2015). Inhibition of Fatty Acid Oxidation Modulates Immunosuppressive Functions of Myeloid-Derived Suppressor Cells and Enhances Cancer Therapies. Cancer Immunol. Res..

[222] Estañ M.C., Calviño E., Calvo S., Guillen-Guio B., Boyano-Adánez M.D.C., De Blas E., Rial E., Aller P. (2014). Apoptotic Efficacy of Etomoxir in Human Acute Myeloid Leukemia Cells. Cooperation with Arsenic Trioxide and Glycolytic Inhibitors, and Regulation by Oxidative Stress and Protein Kinase Activities. PLoS ONE.

[223] Pike L.S., Smift A.L., Croteau N.J., Ferrick D.A., Wu M. (2011). Inhibition of fatty acid oxidation by etomoxir impairs NADPH production and increases reactive oxygen species resulting in ATP depletion and cell death in human glioblastoma cells. Biochim. et Biophys. Acta (BBA)-Bioenerg..

[224] Dheeraj A., Agarwal C., Schlaepfer I.R., Raben D., Singh R., Agarwal R., Deep G. (2018). A novel approach to target hypoxic cancer cells via combining beta-oxidation inhibitor etomoxir with radiation. Hypoxia (Auckl.).

